# Combining novel feature selection strategy and hyperspectral vegetation indices to predict crop yield

**DOI:** 10.1186/s13007-022-00949-0

**Published:** 2022-11-08

**Authors:** Shuaipeng Fei, Lei Li, Zhiguo Han, Zhen Chen, Yonggui Xiao

**Affiliations:** 1 Institute of Farmland Irrigation, Chinese Academy of Agricultural Sciences, Xinxiang, 453002 China; 2 National Wheat Improvement Centre, Institute of Crop Sciences, Chinese Academy of Agricultural Sciences, Beijing, 100081 China; 3 PhenoTrait Laboratory, PhenoTrait Technology Co. Ltd, Beijing, 100096 China

**Keywords:** Wheat yield, Hyperspectral, Vegetation index, Deep neural network, Feature selection

## Abstract

**Background:**

Wheat is an important food crop globally, and timely prediction of wheat yield in breeding efforts can improve selection efficiency. Traditional yield prediction method based on secondary traits is time-consuming, costly, and destructive. It is urgent to develop innovative methods to improve selection efficiency and accelerate genetic gains in the breeding cycle.

**Results:**

Crop yield prediction using remote sensing has gained popularity in recent years. This paper proposed a novel ensemble feature selection (EFS) method to improve yield prediction from hyperspectral data. For this, 207 wheat cultivars and breeding lines were grown under full and limited irrigation treatments respectively, and their canopy hyperspectral reflectance was measured at the flowering, early grain filling (EGF), mid grain filling (MGF), and late grain filling (LGF) stages. Then, 115 vegetation indices were extracted from the hyperspectral reflectance and combined with four feature selection methods, i.e., mean decrease impurity (MDI), Boruta, FeaLect, and RReliefF to train deep neural network (DNN) models for yield prediction. Next, a learning framework was developed by combining the predicted values of the selected and the full features using multiple linear regression (MLR). The results show that the selected features contributed to higher yield prediction accuracy than the full features, and the MDI method performed well across growth stages, with a mean *R*^2^ ranging from 0.634 to 0.666 (mean RMSE = 0.926–0.967 t ha^−1^). Also, the proposed EFS method outperformed all the individual feature selection methods across growth stages, with a mean *R*^2^ ranging from 0.648 to 0.679 (mean RMSE = 0.911–0.950 t ha^−1^).

**Conclusions:**

The proposed EFS method can improve grain yield prediction from hyperspectral data and can be used to assist wheat breeders in earlier decision-making.

**Supplementary Information:**

The online version contains supplementary material available at 10.1186/s13007-022-00949-0.

## Background

Under climate change and global population growth, declining crop yields are putting the global food supply at risk [1, 2]. The development of many superior resistant plant varieties through breeding efforts is an immediate solution. Improving yields is the primary goal of crop breeding programs [3]. However, yield is influenced by both quantitative and qualitative traits, and measuring yield in a large breeding population consisting of thousands of genotypes can be time-consuming and laborious [3–5]. Secondary traits can help breeders predict grain yield at early stages to reduce the time and cost [6], but the traditional manual trait survey methods are not efficient. In recent years, developments in remote sensing and spectroscopy sensor technologies have facilitated the establishment of low-cost, high-throughput phenotyping platforms that can collect large amounts of data related to yield at different stages under various growth environments in breeding efforts [4, 6–8].

The spectroscopy of agriculture can measure different wavelengths of electromagnetic energy interacting with different parts of a growing plant [8]. The goal of spectral science is to quantify phenotypes through interactions between light and plants, such as reflected, absorbed, transmitted, and/or emitted photons [8]. The commonly used sensors in precision agriculture include hyperspectral, RGB, multispectral, and thermal infrared [9–12]. Compared to other sensors, hyperspectral sensors cover many continuous bands, and they have been applied to estimate various crop parameters including yield, biomass, leaf area index (LAI), and chlorophyll content [13–15]. In addition to the raw bands, hyperspectral data can be also derived from integer and fractional-order derivatives to reveal hidden information related to crop growth [15, 16]. The reflectance of electromagnetic energy at different wavelengths is usually summarized as the vegetation index [8], and it is further adopted to predict the physiological properties and agronomic traits of plants. Related studies combined a large number of vegetation indices in various wavelength regions to evaluate crop parameters [15, 17], and different vegetation indices can complement each other to provide more information related to plant growth.

The parsing of large data sets acquired by high-throughput phenotyping platforms requires intensive computational and statistical analysis, which is a challenge for plant breeding programs [18]. Nowadays, many statistical and machine learning-based regression techniques such as support vector regression, random forest regression and extreme learning machine have been applied to build predictive models of plant traits and achieve accurate predictions [15, 19–21]. As a subfield of machine learning, deep learning can automatically learn data representations with multi-layer architecture. The architecture supports complex nonlinear functions, which are learned from the hierarchical output of the previous layers [22]. Deep learning methods such as convolutional neural network , deep neural network (DNN), and residual neural network have achieved high accuracy in various regression and classification tasks in the field of precision agriculture [23–25].

To obtain accurate yield predictions and avoid model overfitting, machine learning algorithms often use feature selection methods to reduce the redundancy of the data [26]. Feature selection methods such as recursive feature elimination, Pearson correlation coefficient, random forest-based mean decrease impurity, and partial least squares based variable importance in the projection have been used to estimate crop parameters ranging from alfalfa and soybean yield prediction to sorghum leaf chlorophyll concentration estimation [3, 16, 17]. Each feature selection method has its unique focus, and most studies have utilized only a single feature selection method for modeling, which inspires this study to combine the characteristics of multiple feature selection methods. Ensemble learning such as stacking regression has gained a lot of attention in the machine learning community. Ensemble learning achieves higher accuracy than base learners in the analysis of hyperspectral data. For the regression, the prediction accuracy of alfalfa yield was improved by combining stacking regression and hyperspectral vegetation indices and reflectance [17]. For hyperspectral image classification, both tangent space collaborative representation classification (TCRC)-bagging and TCRC-boosting ensemble methods outperform the individual classifier [27]. In addition, the deep ensemble method in classification and unmixing experiments of hyperspectral data outperform base spectral and spectral-spatial deep models and classical ensembles employing voting and averaging as a fusing scheme [28]. The above studies demonstrate the superiority of the ensemble approach in processing hyperspectral data. Generally, higher heterogeneity among base learners helps to improve the accuracy of ensemble models [29]. Similarly, there are differences in the features selected by various feature selection methods, resulting in heterogeneity among the output predictions. Therefore, combining multiple feature selection methods in an ensemble pattern has the potential to obtain higher prediction accuracy than individual feature selection methods and full features.

Based on the above descriptions, this study aim to (1) explore the potential application of hyperspectral vegetation indices and DNN in predicting wheat yield; (2) compare the yield prediction accuracy of individual feature selection methods and propose an ensemble feature selection (EFS) method; (3) identify the optimal stage for acquiring hyperspectral data at late wheat growth.

## Materials and methods

### Experimental design

**Fig. 1:**
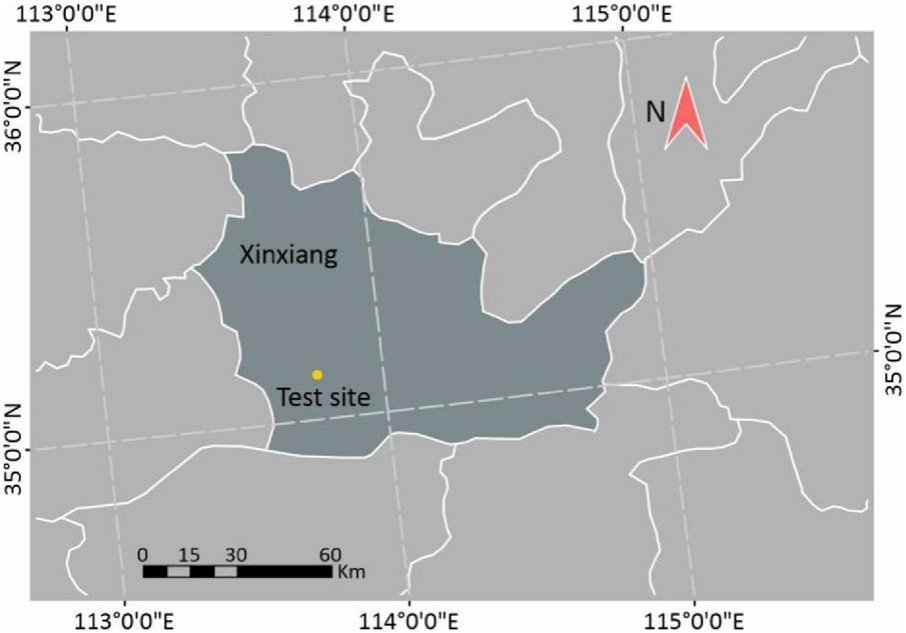
Test site location

**Fig. 2:**
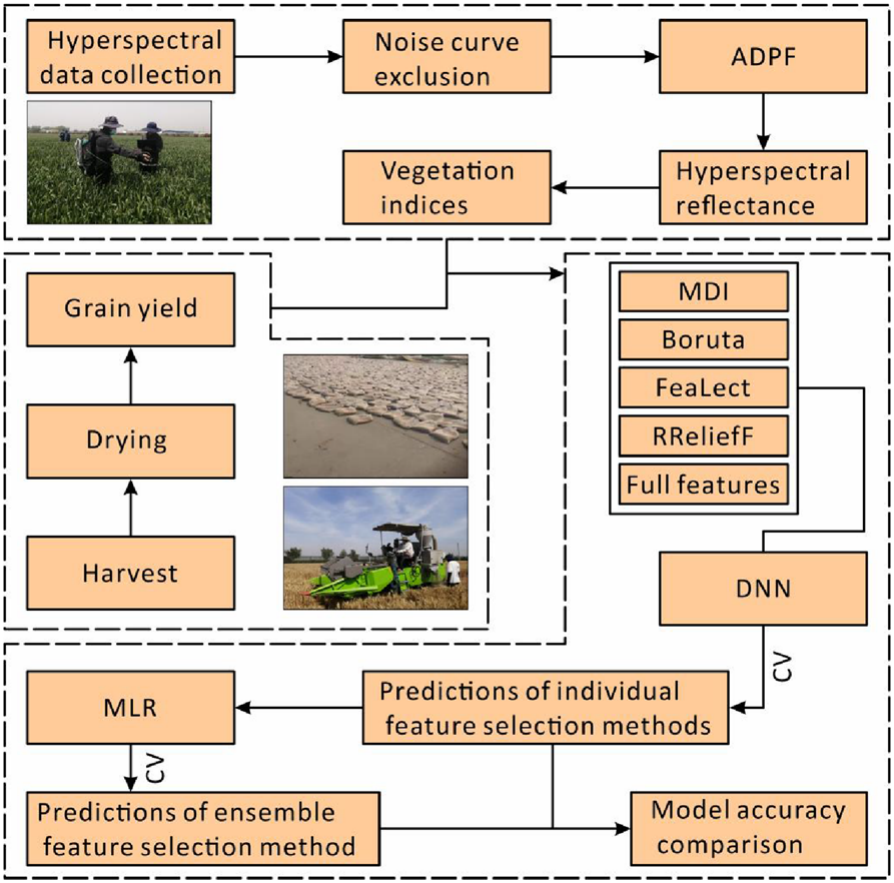
Overall workflow of this study. *ADPF* adaptive degree polynomial filter, *CV* cross-validation, *DNN* deep neural network, *MDI* mean decrease impurity, *MLR* multiple linear regression

This study adopted a panel of 207 varieties. During the growing season in 2018–2019, all varieties were cultivated at the research station of the Chinese Academy of Agricultural Sciences (CAAS) at Xinxiang (35°18′N, 113°51′E; Henan Province, China) (Fig. 1) under two water irrigation levels, namely full and limited irrigation. The field experiments were set up in randomized complete blocks with two replications. Each plot was 4.2 m^2^ in size, with a dimension of 3 m × 1.4 m and six rows with a spacing of 0.2 m. Both irrigation treatments were irrigated with the same amount of water (250 mm) at the tillering stage, while irrigation was continued for full irrigation treatment at the early jointing, heading, and early grain filling stages. The application of fertilizer was optimized based on the soil conditions in the area. All plants were harvested at physiological maturity using the combined harvester. The grain yield was measured at a grain moisture level of 12.5%. The workflow of this study is shown in Fig. 2.

### Hyperspectral data acquisition and processing

A high-spectral-resolution spectrometer (Fieldspec 3, Analytical Spectral Devices ASD, Boulder, CO, USA) connected with a 25° field of view fiber optic was used to collect the canopy reflectance of each plot from 350 to 2500 nm. The visible-to-near-infrared range (350–1000 nm) had a spectral resolution of 3 nm, while the shortwave infrared region had a spectral resolution of 10 nm (1000–2500 nm). The sensor was placed 100 cm above the canopy in a nadir position and operated vertically. The canopy reflectance was measured at four separate sites in each plot between 11:00 a.m. and 1:00 p.m. local time on a clear day. For each site, ten readings were taken, and the average of these 40 readings was taken to calculate the canopy reflectance of the plot. Before measuring canopy reflectance, a BaSO4 calibration panel was used to estimate the incoming radiation and reflectance. This processing was conducted every ten plots. Then, spectral measurements were carried out at flowering, early grain filling (EGF), mid grain filling (MGF), and late grain filling (LGF) stages. The View Spec software (ASD Inc, Boulder, CO, USA) was employed to eliminate noise from spectral curves, calculate the average of numerous spectral curves, and generate a reflectance file. To eliminate noise during the spectrum collection process, the adaptive degree polynomial filter (ADPF) was used [30]. ADPF adds a statistical heuristic to the Savitzky–Golay method to improve signal fidelity while reducing statistical noise. Following filtering, a database of 115 vegetation indices (Additional file 1: Table S1) was established as input features to the yield prediction model.

### Deep neural network

**Fig. 3:**
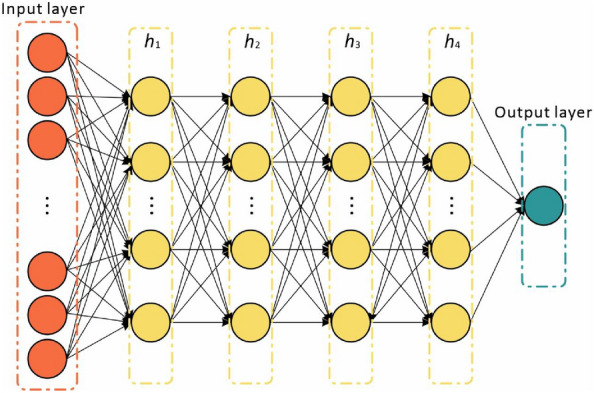
A schematic diagram of the deep neural work. *h* hidden layers

In this study, the fully-connected feedforward DNN based on a multi-layer artificial neural network was used to analyze the effectiveness of the proposed EFS method, which has been applied to solve various machine learning problems [22–24]. This study designed a fully-connected input layer and multiple hidden layers and connected them to a final fully connected layer for the final regression to predict the grain yield (Fig. 3). A detailed description of DNN can refer to [31]. Search for appropriate hyperparameters is a key step in the implementation of DNN models. The hyperparameters (Table 1) were tuned for DNN by performing a grid search with tenfold cross-validation on the training dataset. The DNN model was implemented in R software using the H2O package (https://CRAN.R-project.org/package=h2o).

**Table 1:** Deep neural network hyperparameter tuning and the range for each hyperparameter

Model parameters	Value
Units	From 50 to 150 by a step of 10
Epochs	10
Hidden layers	3, 4, 5, and 6
Learning rate	0.005
Loss function	Automatic setting
Regularization method	Dropout
Activation function	Rectified linear activation unit function

### Feature selection method

In this study, four feature selection methods, namely MDI, Boruta, FeaLect, and RReliefF, were used to verify the effectiveness of the proposed EFS method. The EFS method is based on the idea of ensemble learning. The four feature selection methods have different principles, and they have achieved satisfactory accuracy in previous studies [14, 16, 32, 33], which is in line with the principles of diversity and adequacy of ensemble learning [29].

### Mean decrease impurity

MDI is a random forest-based feature selection method. The random forest utilizes randomized decision trees and impurity measurements to calculate the importance of various features [34]. When the random forest employs the Gini index as its impurity measurement, one such technique is referred to as MDI. Breiman [34] proposed to estimate the importance of a variable *k* for predicting *y* (i.e., grain yield) by cumulating the weighted impurity decreases (*p*(*t*) *∆i*(*s*_*t*_*, t*)) for all nodes *t*, and *k* is used and averaged over all *N*_*T*_ trees in the forest in the following equation:

1 $$MD{I}_{k}=\frac{1}{{N}_{T}}{\sum }_{T} {\sum }_{t\in T:v\left({s}_{t}\right)=k} p(t)\Delta i\left({s}_{t},t\right)$$

where *p*(*t*) represents the proportion *Nt/N* of a sample reaching *t*, and *v(s*_*t*_*)* represents the variable used in split *s*_*t*_.

### Boruta

The Boruta algorithm is an extension of the idea proposed by [35]. Boruta calculates the Z-scores for each input feature concerning the shading attribute [36]. In this study, the ranking of each vegetation index was determined based on its Z-score. The Z-score was calculated as follows [36]. First, generate a randomly ordered duplicate variable ${x}_{t}^{\mathrm{^{\prime}}}$ for a particular input vector, ${x}_{v}$ for increasing randomness and eliminating the correlations between duplicate predictors and targets $\left({y}_{t}\right)$ for a group of discrete inputs $\left({x}_{t}\in {R}^{n}\right)$, *T* and a target variable $\left({y}_{t}\in {\varvec{R}}\right)$ with several inputs (*n*) and *t* = 1, 2, …*T*. Then, use the random forest algorithm to predict the target (${y}_{t}$) with the duplicated input (${x}_{t}^{\mathrm{^{\prime}}}$) and actual input (${x}_{t}$). Finally, the variance importance measure, i.e., permutation importance or mean decrease accuracy (MDA) is calculated for each input ${x}_{t}$ and respective shadow input (${x}_{t}^{\mathrm{^{\prime}}}$) overall trees as follows:

2 $$MDA=\frac{1}{{m}_{\text{tree }}}{\sum }_{m=1}^{{m}_{\text{ree }}} \frac{{\sum }_{t\in OOB} I\left({y}_{t}=f\left({x}_{t}\right)\right)-{\sum }_{t\in OOB} I\left({y}_{t}=f\left({x}_{t}^{n}\right)\right)}{|OOB|}$$

where *I*(∙) represents the indicator function; OOB represents the prediction error of each training sample based on bootstrap aggregation; ${y}_{t}=f\left({x}_{t}\right)$ represents predicted values before permuting; and ${y}_{t}=f\left({x}_{t}^{n}\right)$ represents predicted values after permuting. The Z-scores are calculated as:

3 $$Z-\mathrm{ score }=\frac{MDA}{SD}$$

where SD is the standard deviation of accuracy losses.

### FeaLect

FeaLect is a feature selection method proposed by [32] based on combinatorial analysis of least absolute shrinkage and selection operator (LASSO). Let *B* be a random sample of size *m*, and it is generated by selecting without replacement from the given training data *D*, where *n* =|*D*| and $\gamma \in (\mathrm{0,1})$ represents a parameter that controls the size of the sample set. The Lars algorithm is applied to recover the entire regularization path using the training set *B*. Let ${F}_{k}^{B}$ be a set of selected features by the LASSO when λ allows the selection of *k* features. The number of selected features is decreasing in λ, and we have:

4 $$\mathrm{\varnothing }={F}_{0}^{B}\subset \dots {F}_{k}^{B}\subset {F}_{k+1}^{B}\subset \cdots \subset {F}_{d}^{B}=F.$$

For each feature *f*, a scoring mechanism was defined based on whether it is selected in ${F}_{k}^{B}$:

5 $${S}_{k}^{B}(f):=\left\{\begin{array}{l}\frac{1}{k}\text{ if }f\in {F}_{k}^{B}\\ 0\mathrm{ otherwise }\end{array},\right.$$

The above randomized process was randomly cycled several times for various random subsets *B* to calculate the average score of *f* when *k* features were selected. Then, the sum of average scores was used to calculate the total score for each feature:

6 $$S\left(f\right):={\sum }_{k}{\mathbb{E}}_{B}\left[{S}_{k}^{B}\left(f\right)\right].$$

For features with a score of 0, the FeaLect program was rerun on them to ensure that the relative importance among all features was determined.

### RReliefF

The RReliefF algorithm is an improvement on Relief [37]. It can solve noisy multi-class problems and regression problems and can handle incomplete data. RReliefF introduces probabilities that can be modeled by the relative distance between the predicted values of two observations, thus allowing to calculate the weights of features [14]. The pseudo code of RReliefF algorithm is shown in Algorithm 1.
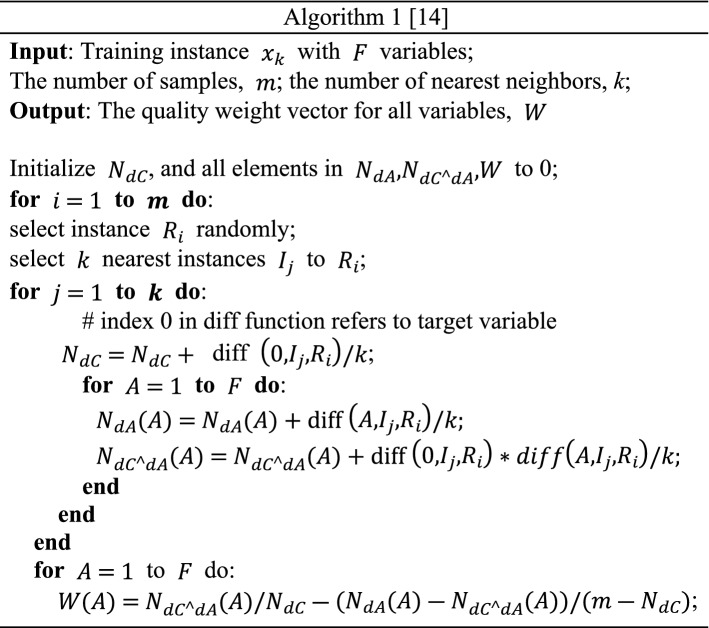


where $\mathrm{diff}\left(A,{I}_{j},{R}_{i}\right)=\frac{\mid \mathrm{ value }\left(A,{I}_{j}\right)-\mathrm{value}\left(A,{R}_{i}\right)\mid }{\left({A}_{\mathrm{max}}-{A}_{\mathrm{min}}\right)}$, value $\left(A,{I}_{j}\right)$ is the value of *A* attributes for samples ${I}_{j}$ and ${R}_{i}$, and *A*_max_ and *A*_min_ are respectively the maximum and minimum values of variable *A* for *m* samples.

### Ensemble feature selection method

Instead of using a single machine learning method, ensemble learning builds and combines multiple learners to accomplish the learning task. This study referred to the idea of ensemble learning to combine models constructed by different feature selection methods to verify whether the EFS method can achieve better model performance than individual feature selection methods.

**Fig. 4:**
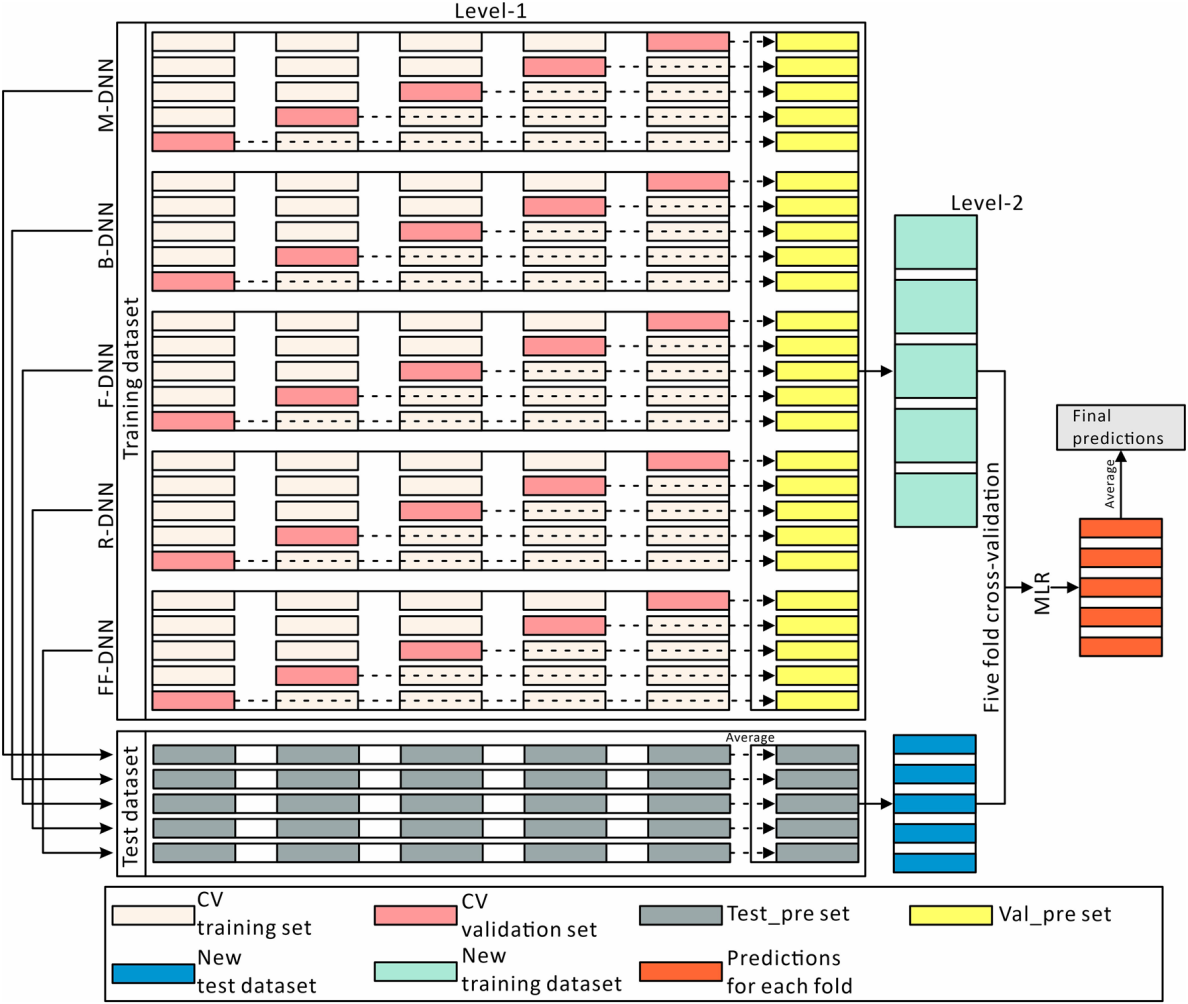
Structure of the proposed ensemble feature selection framework. *B* Boruta, *CV* cross-validation, *DNN* deep neural network, *F* FeaLect, *FF* full features, *M* mean decrease impurity, *MLR* multiple linear regression, *R* RReliefF

First, according to the importance ranking of each feature selection method, features were input to the DNN in turn until the training error reached the minimum. At this time, the input features were considered the optimal feature combination. Then, the four optimal feature combinations obtained by the four feature selection methods and full features were taken to train five DNN models. Finally, the predictive capability of these five models was combined through a modeling framework similar to stacking ensemble learning [38]. As shown in Fig. 4, the steps for the proposed EFS method are as follows:

- The original datasets of each feature selection method and full features are divided into a training dataset and a test dataset at the ratio of 4:1;
- For each DNN model at level-1, fivefold cross-validation without stratification is performed to train and output the val_pre dataset for the validation dataset and test_pre set for the test dataset in each fold. The val_pre datasets are combined as the new training dataset and the test_pre sets are averaged as the new test set;
- The new training set is adopted to train the final model by multiple linear regression (MLR) at level-2 through fivefold cross-validation. The final prediction for each fold on the new test dataset are output and then averaged to obtain the final prediction.

In this study, the above process was repeated 20 times, and the average accuracy parameters of 100 tests generated in the cross-validation process were used to evaluate the model performance.

### Model accuracy assessment parameters

Coefficient of determination (*R*^2^) and root mean square error (RMSE) were used to evaluate the accuracy of the yield prediction model. The calculation of *R*^2^ and RMSE are as follows:

7 $$R^{2} = 1 - \frac{{\sum\nolimits_{i = 1}^{n} {\left( {\hat{y}_{i} - y_{i} } \right)^{2} } }}{{\sum\nolimits_{i = 1}^{n} {\left( {y_{i} - \overline{y}} \right)^{2} } }}$$

8 $$\mathrm{RMSE}=\sqrt{\frac{{\sum }_{i=1}^{n} {\left({\widehat{y}}_{l}-{y}_{i}\right)}^{2}}{n}}$$

where ${y}_{i}$ and ${\widehat{y}}_{i}$ are the measured and the predicted grain yield, respectively; $\overline{y}$ is the mean of the measured grain yield, and *n* is the total number of testing samples. A larger *R*^2^ and a smaller RMSE indicate a stronger predictive capability of the model.

### Statistical analysis

A mixed linear model was adopted to test the significance of variation between genotypes, irrigation treatments, and their interactions for the measured and predicted grain yield. The equation of the model is as follows [39]:

9 $$Y=X\beta +Z\mu +\varepsilon ,$$

where *Y* is the response demonstrated by fixed effect (*β*) and random effect (*µ*) with a random error (*ε*); *X* and *Z* denote fixed and random effects, respectively. Broad-sense heritability refers to the percentage of genetic variation to the total variation in the phenotype, with a value between 0 and 1. The heritability of 0 and 1 indicate that the phenotypic variation is entirely influenced by environmental and genetic factors, respectively. The heritability was calculated by the following formula:

10 $${H}^{2}={\sigma }_{\mathrm{g}}^{2}/\left({\sigma }_{\mathrm{g}}^{2}+{\sigma }_{\varepsilon }^{2}/r\right),$$

where *r* represents the number of replications per treatment; σ_g_^2^ and σ_ε_^2^ are the genotypic and error variances, respectively.

## Results

### Descriptive statistics of measured wheat yields

**Fig. 5:**
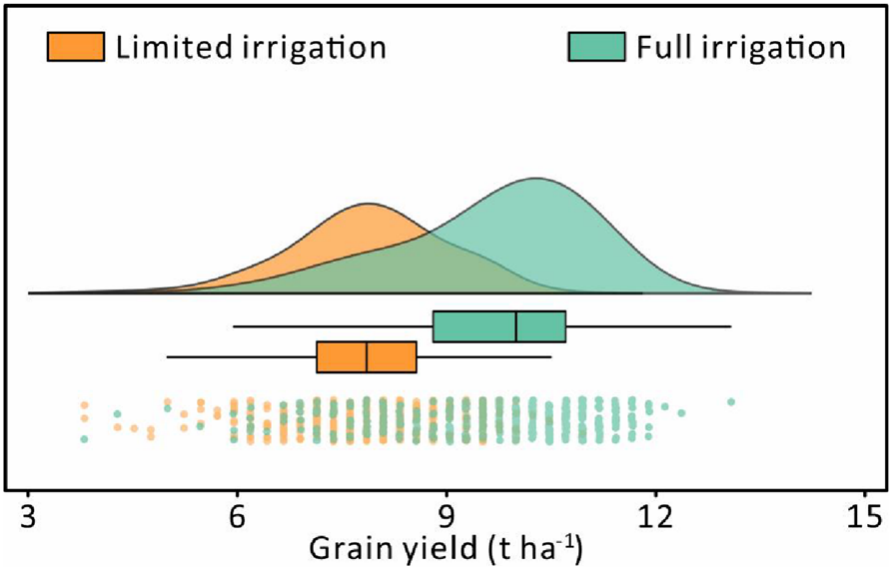
Distribution of wheat yield under full and limited irrigation treatments

The descriptive statistics and distribution of measured yields from both irrigation treatments are shown in Fig. 5. The resulting mean yield values for the full and limited irrigation treatments were 9.64 and 7.79 t ha^−1^, respectively. Compared to the limited irrigation treatment (1.11 t ha^−1^), the yield under full irrigation treatment had a wider range of distribution with a higher standard deviation (1.43 t ha^−1^). Meanwhile, the results of the Shapiro–Wilk test (*P* ≤ 0.05) indicated that the yields under both irrigation treatments were normally distributed.

### Feature selection results

The 115 vegetation indices were ranked according to the results of the MDI, Boruta, FeaLect, and RReliefF methods, respectively. The detailed ranking of vegetation indices is shown in Additional file 1: Table S2–S5. The results show that there are differences in the ranking of vegetation indices among the four feature selection methods. Meanwhile, the performance of some vegetation indices was stable and excellent. For example, the Datt8, PWI, Datt7, DPI, PRI, PRI_norm, mREIP, and REP_Li ranked in the top 30 for all four feature selection methods at the flowering stage (Additional file 1: Table S2–S5). These better-performing indices form the basis for the model’s outperformance.

**Fig. 6:**
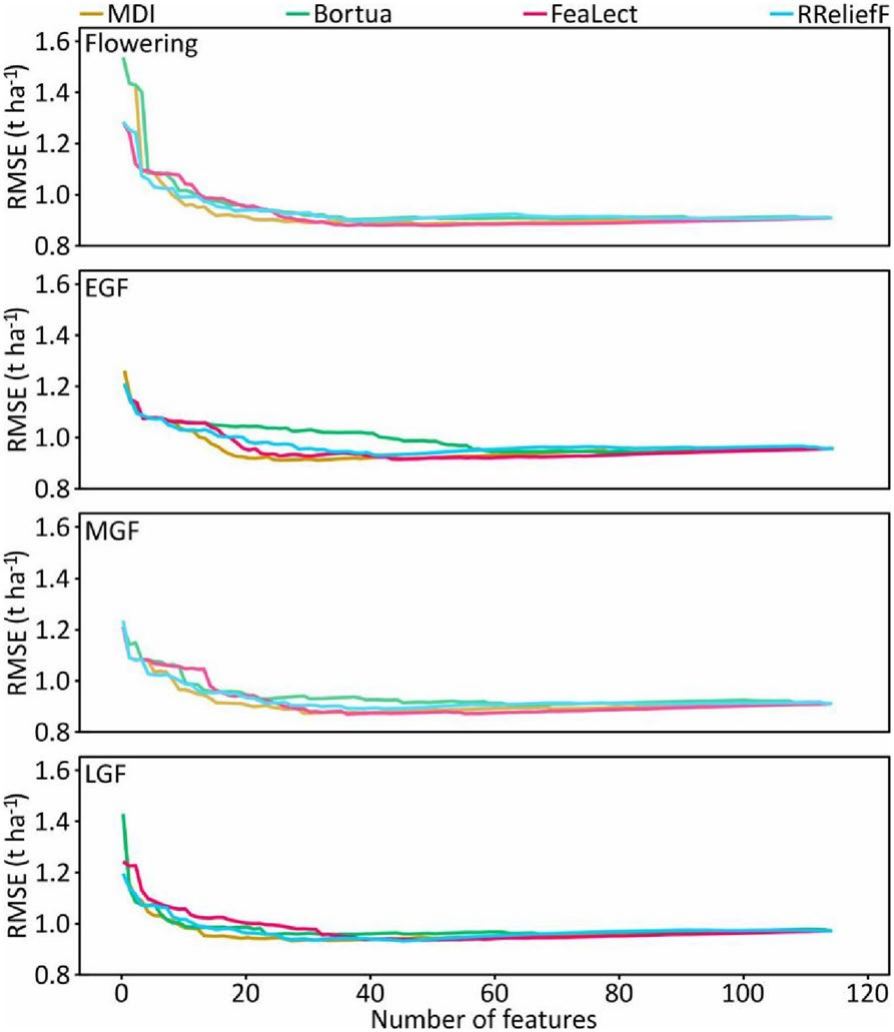
The training accuracy of the deep neural network model as a function of the number of features. *EGF* early grain filling, *MGF* mid grain filling, *LGF* late grain filling, *MDI* mean decrease impurity

**Fig. 7:**
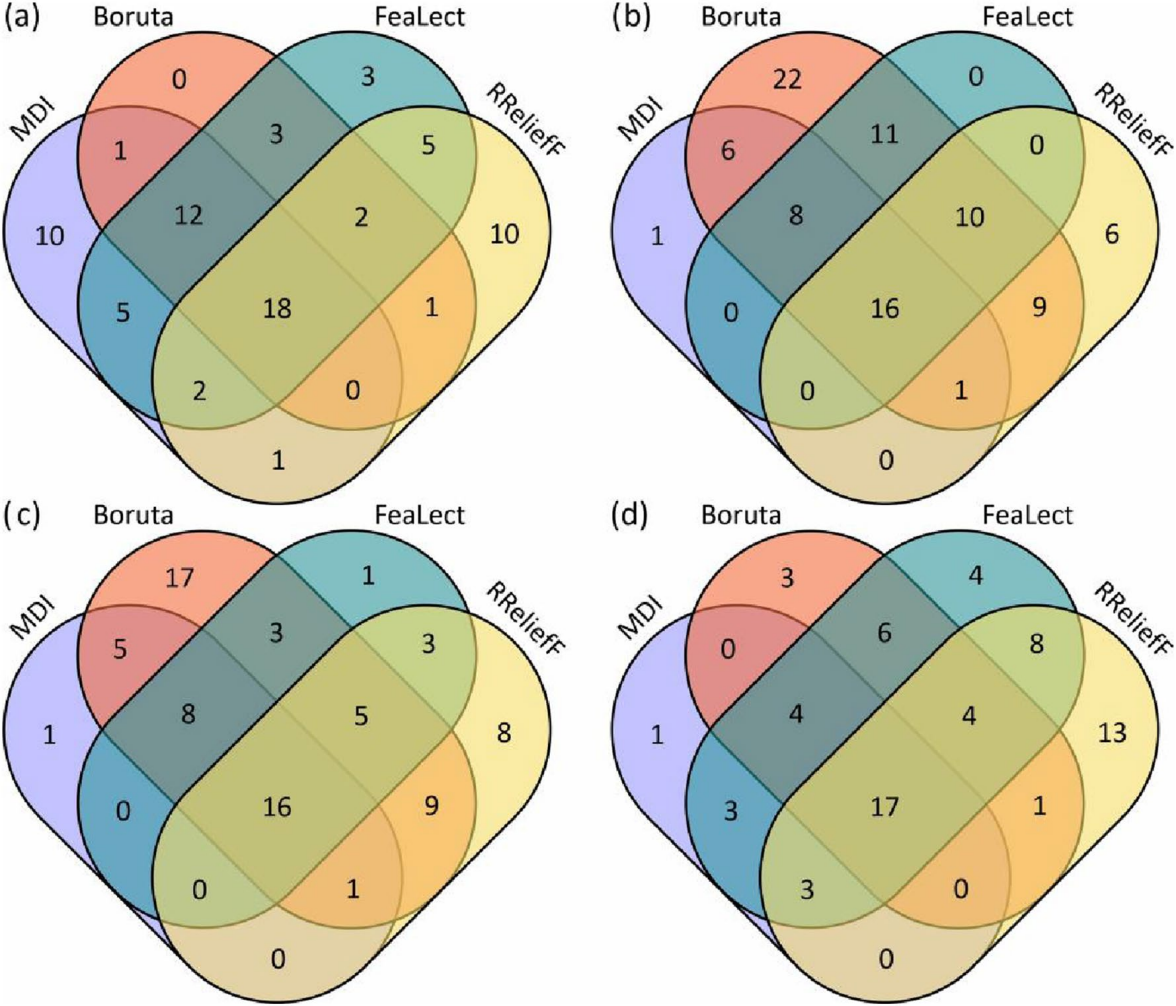
The Venn diagram of the selected features for each feature selection method. **a** flowering, **b** early grain filling, **c** mid grain filling, and **d** late grain filling. *MDI* mean decreasing impurity

Vegetation indices were added to the DNN model in turn according to the ranking result, and the model training error (RMSE) was updated until all 115 indices were included to further investigate the features with superior performance. Note that in this procedure, the default hyperparameters of DNN in the h2o.deeplearning function were used to improve the efficiency of feature selection. With the input of more features, the training error of the DNN model first declined to the minimum and then slightly increased (Fig. 6). The training error of the MDI method was the lowest at all measured stages. Furthermore, the MDI method achieved the lowest error with the minimum number of features at the early, mid, and late grain filling stages. The performances of the remaining feature selection methods varied across the stages. For each feature selection method, the number of features that contributed to the lowest training error was selected to develop the ensemble feature selection model. The Venn diagram (Fig. 7) was used to represent the number of features that are common and unique to multiple feature selection methods.

### Performance of yield prediction model

**Fig. 8:**
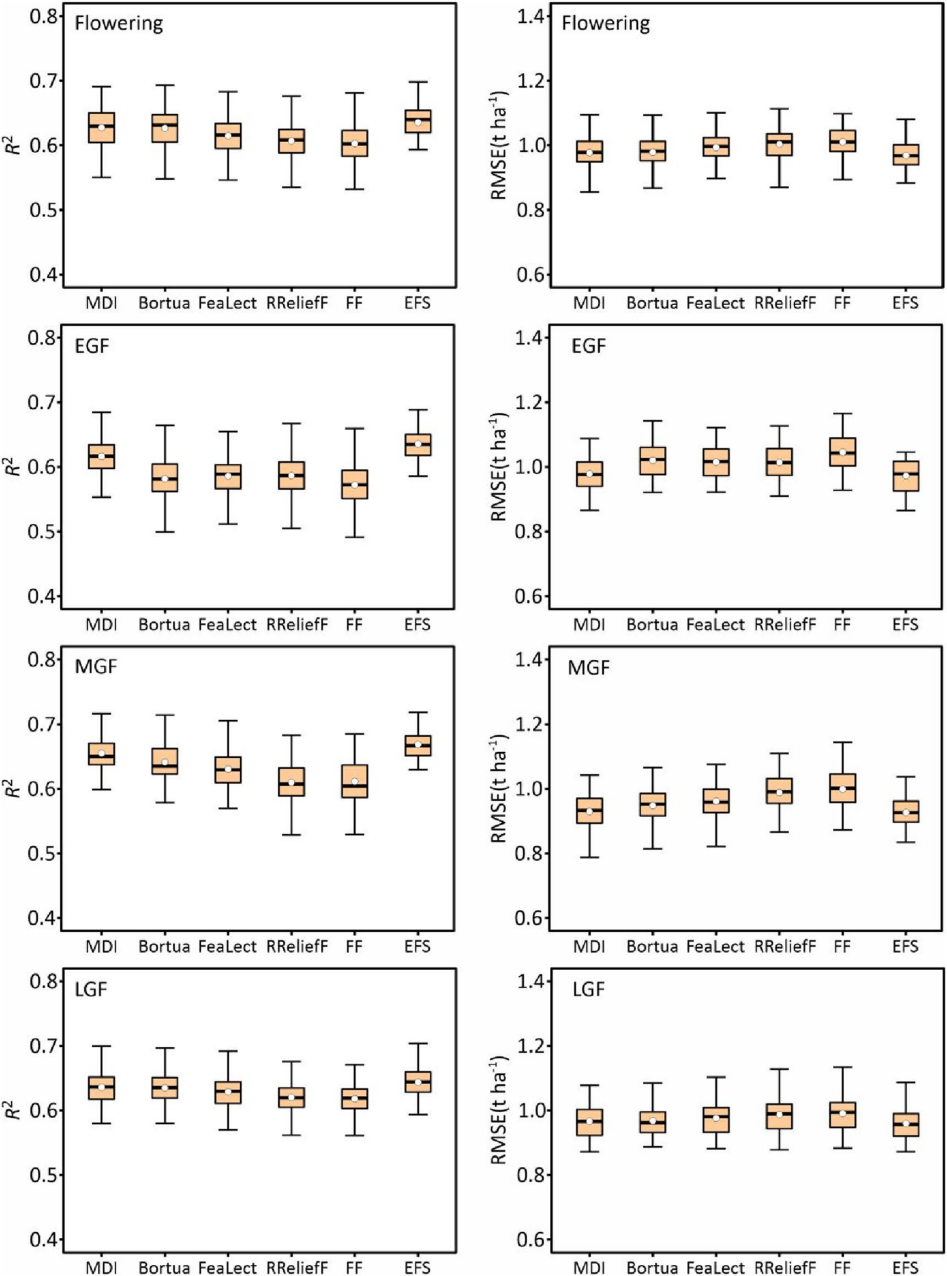
The statistical distributions of the yield prediction accuracy of various feature selection methods. *EGF* early grain filling, *MGF* mid grain filling, *LGF* late grain filling, *MDI* mean decrease impurity, *FF* full features, *EFS* ensemble feature selection

To validate the model adaptability, the prediction accuracy based on the selected features, full features, and the EFS method on the test set was analyzed, the accuracy statistics are illustrated in Fig. 8. As for the feature selection methods at the flowering stage, the MDI method yielded the highest mean *R*^2^ of 0.636 (mean RMSE = 0.964 t ha^−1^), followed by the Boruta method (mean *R*^2^ = 0.627, mean RMSE = 0.977 t ha^−1^) and the FeaLect method (mean *R*^2^ = 0.617, mean RMSE = 0.990 t ha^−1^). Compared to full features (mean *R*^2^ = 0.604, mean RMSE = 1.006 t ha^−1^), the prediction accuracy based on the RReliefF method was slightly lower (mean *R*^2^ = 0.589, mean RMSE = 1.030 t ha^−1^). The best predictive performance for the EGF stage was achieved by the MDI method with a mean *R*^2^ of 0.634 and an RMSE of 0.967 t ha^−1^, followed by the Boruta method (mean *R*^2^ = 0.612, mean RMSE = 0.995 t ha^−1^). Also, the FeaLect method (mean *R*^2^ = 0.608, mean RMSE = 1.001 t ha^−1^) obtained a similar predictive result to the RReliefF method (mean *R*^2^ = 0.607, mean RMSE = 1.003 t ha^−1^). Besides, full features yielded the lowest mean *R*^2^ of 0.570 (mean RMSE = 1.046 t ha^−1^). The best predictive performance for the MGF stage was also achieved by the MDI method (mean *R*^2^ = 0.666, mean RMSE = 0.926 t ha^−1^), followed by the Boruta method (mean *R*^2^ = 0.658, mean RMSE = 0.938 t ha^−1^), the RReliefF method (mean *R*^2^ = 0.643, mean RMSE = 0.958 t ha^−1^), the FeaLect method (mean *R*^2^ = 0.639, mean RMSE = 0.963 t ha^−1^), and full features (mean *R*^2^ = 0.616, mean RMSE = 0.992 t ha^−1^). Different from the first three stages, at the LGF stage, the Boruta method achieved the highest prediction accuracy (mean *R*^2^ = 0.643, mean RMSE = 0.957 t ha^−1^), followed by the MDI method (mean *R*^2^ = 0.639, mean RMSE = 0.962 t ha^−1^). The prediction performance of the FeaLect method (mean *R*^2^ = 0.624, mean RMSE = 0.982 t ha^−1^), the RReliefF method (mean *R*^2^ = 0.627, mean RMSE = 0.979 t ha^−1^) and full features (mean *R*^2^ = 0.622, mean RMSE = 0.985 t ha^−1^) was similar.

**Fig. 9:**
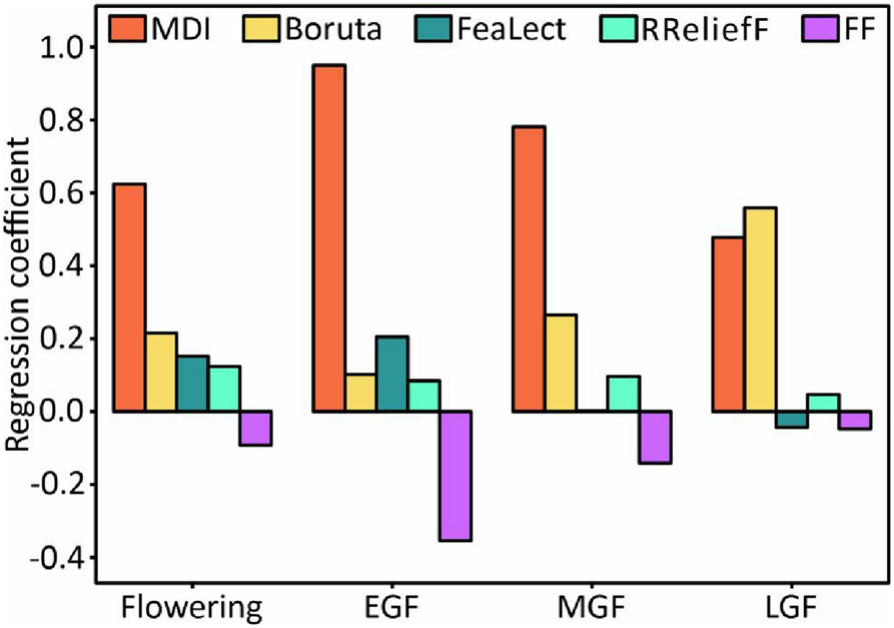
The mean value of regression coefficients assigned to each feature selection method. *EGF* early grain filling, *MGF* mid grain filling, *LGF* late grain filling, *MDI* mean decrease impurity, *FF* full features

Compared to the individual feature selection method with the highest prediction accuracy, the EFS method improved the mean *R*^2^ to 0.648, 0.650, 0.679, and 0.652 respectively for the stages of flowering, EGF, MGF, and LGF, and the values of RMSE decreased. In the EFS method, MDI contributes more, and the regression coefficients assigned within MLR were higher in all periods (Fig. 9).

### Analysis of yield prediction value

**Fig. 10:**
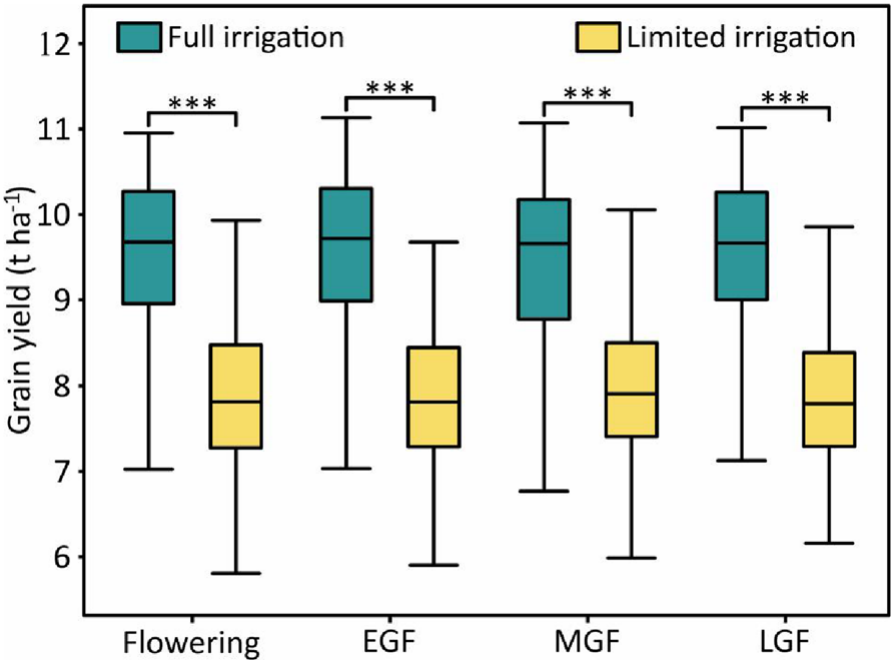
Distribution of yield prediction values output by the ensemble feature selection method. *EGF* early grain filling, *MGF* mid grain filling, *LGF* late grain filling. *** significant at *P* ≤ 0.0001

The predicted wheat yield under both irrigation treatments was output by the EFS method. The wheat yield under full irrigation treatment was significantly higher (*P* ≤ 0.0001) than that under limited irrigation treatment for all measured stages (Fig. 10). ANOVA (Table 2) revealed that genotypes, treatments, and the interactions of genotype and treatment had significant effects on the predicted yield for all measured stages, which was consistent with the measured yield. Similar to the measured yield (*H*^2^ = 0.63), the *H*^2^ of the predicted yield was high, with the value of 0.73, 0.71, 0.77, and 0.62 for the stages of flowering, EGF, MGF, and LGF respectively under the two irrigation treatments, suggesting that most of the phenotypic variation was determined by genetic factors.

**Table 2:** Analysis of variance for the predicted grain yield output by the ensemble feature selection method and the measured grain yield

Grain yield	F-value			*H*^2^
	Genotype (G)	Treatment (T)	G × T interaction	
Flowering	5.27***	1789.08***	1.59***	0.73
EGF	5.43***	1487.70***	1.80***	0.71
MGF	6.60***	2334.92***	1.68***	0.77
LGF	4.64***	2050.94***	2.23***	0.62
Measured	3.43***	781.97***	1.44***	0.63

*EGF* early grain filling, *MGF* mid grain filling, *LGF* late grain filling

*** significant at *P* ≤ 0.0001

## Discussion

The application of canopy hyperspectral data to predict crop yields in precision agriculture management is not new [3, 14, 17]. However, the similarity of traits among many breeding lines will lead to a heavy workload and make it difficult to perform accurate monitoring [40]. The traditional method of collecting phenotypes reduces the efficiency of selecting superior breeding varieties [11]. Hyperspectral remote sensing method with high spectral resolution can obtain continuous and fine spectral profiles of terrestrial objects at wide-range wavelengths [41]. Compared with RGB and multispectral data, hyperspectral data contains rich information related to plant growth and can help to detect minor differences between various breeding varieties [11].

Previous studies have shown that the full bands of hyperspectral reflectance contribute higher yield prediction accuracy than the vegetation index set [8, 14], but the ultra-high dimensionality of the full bands makes the program run much longer. Considering the strong collinearity and information overlap among a large number of vegetation indices composed of hyperspectral data, choosing appropriate input features plays an important role in reducing the dimensionality and improving the prediction accuracy. Recent developments [42, 43] in hyperspectral image analysis based on deep learning and feature selection have inspired us to develop improved feature selection algorithms for crop yield evaluation using hyperspectral vegetation indices. In this study, four feature selection methods and a newly proposed EFS method were applied. MDI has been widely used for crop phenotype assessment [16, 24], the results showed that the MDI method performed better among the four feature selection methods at all growth stages. The MDI score was calculated based on node impurity, which measures the homogeneity of the variable [16]. Our selected vegetation indices use a wide band interval, which makes vegetation indices vary widely from each other. In addition, since MDI is a tree-based scoring measure, the problem of multicollinearity is avoided, and the selected features are simple and efficient [16]. Compared to MDI, only a few studies have investigated the effectiveness of Boruta in estimating crop parameters [33, 36]. The Boruta method in this study showed a predictive performance second only to MDI (at the stages of flowering, EGF, and MGF) or comparable to MDI (at the LGF stage). The excellent performance of MDI and Boruta methods proves the effectiveness of random forest algorithms in selecting features. The FeaLect method has only been used in the diagnosis of lymphoma and has achieved satisfactory classification results [32], and this study is the first to deal with the regression problem. FeaLect obtained better prediction results than RReliefF and full features and showed its potential in estimating crop parameters. Although the RReliefF algorithm performed well in previous report [14], its performance in this study was only higher than the full features. The advantage of the RReliefF method is that it does not require training, which helps to save program execution time. As a deep learning method, DNN has shown high prediction accuracy in evaluating crop yields and has advantages in handling large samples of complex nonlinear data [23]. Although deep learning models are generally considered to be good at extracting information from raw features, this study suggests that feature engineering in deep learning is still beneficial. Previous studies have also performed feature selection by combining remote sensing data and DNN to predict crop yield [24].

Similar to ensemble learning, different feature selection methods may yield feature subsets that can be considered as local optima in the feature subset space, while EFS can combine the local optima in each feature subset to obtain a better model performance. The EFS method proposed by [44] selects the top-ranked features of multiple feature selection methods to improve prediction accuracy. A new EFS method based on stacking regression was proposed in this study. The results showed that the proposed EFS method improved prediction accuracy at all growth stages and made good predictions of wheat yield. Meanwhile, the significant differences between treatments and varieties indicated the practical value of the ensemble method in screening varieties (Table 2). Overall, the newly proposed EFS method maximizes the potential of the huge vegetation index dataset for crop yield predicting, thus enabling the best yield trait-related information to be fully utilized. The unsatisfactory performance of RReliefF inspires future research to combine more model training-based feature selection methods, such as recursive feature elimination and variable importance in projection, to explore a more reasonable combination of the EFS method. To verify the adaptability of the proposed method, experiments will be conducted on other crops for future analyses.

Winter wheat canopy display different structural properties during the growth cycle, which affects the optical signal at different growth stages [45]. Therefore, the difference in collection time may result in some prediction errors of winter wheat yield. The middle and late stages of wheat growth were proven to have higher yield prediction accuracy than the early stages [9, 19]. Our results indicate that the MGF stage achieved the highest prediction accuracy, which helps to save the number of data collection and reduce the cost.

## Conclusions

Pre-harvest insight to yield can help to reduce breeding efforts and optimize field management practices. Remote sensing platforms have been widely used to predict yield, which provides a fast approach for collecting data and reduces labor costs and problems associated with destructive sampling. This study developed an EFS method that combines multiple feature selection methods based on DNN and hyperspectral vegetation indices. The results indicated that the MDI feature selection method performs best in grain yield prediction among the four feature selection methods at most measured stages, followed by Boruta, FeaLect, and RReliefF. The EFS method outperformed all individual feature selection methods, and the highest accuracy was achieved at the MGF stage. Our study demonstrated the efficacy of using hyperspectral vegetation indices and the proposed EFS method for predicting wheat yield. In future work, comprehensive studies will be conducted in different environments to validate the transferability of this EFS method and identify the best combination of feature selection methods.

## Supplementary Information

**Additional file 1: Table S1.** Vegetation indices used in this study. **Table S2.** Vegetation index ranking of feature selection methods at flowering. **Table S3.** Vegetation index ranking of feature selection methods at early grain filling. **Table S4.** Vegetation index ranking of feature selection methods at mid grain filling. **Table S5.** Vegetation index ranking of feature selection methods at mid grain filling.

## Data Availability

The datasets used in this study are available from the corresponding author on reasonable request.
