# Supplementary material for: Combining novel feature selection strategy and hyperspectral vegetation indices to predict crop yield
Source: Plant Methods. 2022 Nov 8;18:119. doi: 10.1186/s13007-022-00949-0 (PMC9641855; doi:10.1186/s13007-022-00949-0)
Supplement: Supplementary file 1 — Additional file 1: Table S1. Vegetation indices used in this study. Table S2. Vegetation index ranking of feature selection methods at flowering. Table S3. Vegetation index ranking of feature selection methods at early grain filling. Table S4. Vegetation index ranking of feature selection methods at mid grain filling. Table S5. Vegetation index ranking of feature selection methods at mid grain filling. [file 13007_2022_949_MOESM1_ESM.docx]

**Table S1** Vegetation indices used in this study.

| Full Form | Formula | Reference |
| --- | --- | --- |
| Boochs | Boochs=$D_{703}$ | [1] |
|  | Boochs2=$D_{720}$ | [1] |
| Cellulose absorption index | CAI=$0.5\cdot\left( R_{2000}+R_{2200} \right)-R_{2100}$ | [2] |
| Chlorophyll absorption ratio index | CARI=$R_{700}\cdot abs\left( a\cdot670+R_{670}+b \right)/R_{670}\cdot\left( a^{2}+1 \right)^{0.5}$  $a=\left( R_{700}-R_{550} \right)/150$ $b=R_{550}-(a\cdot550)$ | [3] |
| Carter | Carter=$R_{695}/R_{420}$ | [4] |
|  | Carter2=$R_{695}/R_{760}$ | [4] |
|  | Carter3=$R_{605}/R_{760}$ | [4] |
|  | Carter4=$R_{710}/R_{760}$ | [4] |
|  | Carter5=$R_{695}/R_{670}$ | [4] |
|  | Carter6=$R_{550}$ | [4] |
| Curvature index | CI=$R_{675}\cdot R_{690}/R_{683}^{2}$ | [5] |
|  | CI2=$R_{760}/R_{700}-1$ | [6] |
| Chlorophyll absorption integra | CAI=$\int_{600nm}^{735nm} R$ | [2] |
| Carotenoid reflectance index | CRI1=$1/R_{515}-1/R_{550}$ | [6] |
|  | CRI2=$1/R_{515}-1/R_{770}$ | [6] |
|  | CRI3=$1/R_{515}-1/R_{550}\cdot R_{770}$ | [6] |
|  | CRI4=$1/R_{515}-1/R_{700}\cdot R_{770}$ | [6] |
| First derivative indices | D1=$D_{730}/D_{706}$ | [5] |
|  | D2=$D_{705}/D_{722}$ |  |
| Datt | Datt=$\left( R_{850}-R_{710} \right)/\left( R_{850}-R_{680} \right)$ | [7] |
|  | Datt2=$R_{850}/R_{710}$ | [7] |
|  | Datt3=$D_{754}/D_{704}$ | [7] |
|  | Datt4=$R_{672}/\left( R_{550}\cdot R_{708} \right)$ | [8] |
|  | Datt5=$R_{672}/R_{550}$ | [8] |
|  | Datt6=$\left( R_{860} \right)/\left( R_{550}\cdot R_{708} \right)$ | [8] |
|  | Datt7=$\left( R_{860}-R_{2218} \right)/\left( R_{860}-R_{1928} \right)$ | [9] |
|  | Datt8=$\left( R_{860}-R_{1788} \right)/\left( R_{860}-R_{1928} \right)$ | [9] |
| Double differene | DD=$\left( R_{749}-R_{720} \right)-\left( R_{701}-R_{672} \right)$ | [10] |
|  | DDn=$2\cdot\left( R_{710}-R_{660}-R_{760} \right)$ | [11] |
| Double-peak optical index | DPI=$\left( D_{688}\cdot D_{710} \right)/D_{697}^{2}$ | [5] |
| Disease-water stress indices | DWSI1=$R_{800}/R_{1660}$ | [12] |
|  | DWSI2=$R_{1660}/R_{550}$ | [12] |
|  | DWSI3=$R_{1660}/R_{680}$ | [12] |
|  | DWSI4=$R_{550}/R_{680}$ | [12] |
|  | DWSI5=$\left( R_{800}+R_{550} \right)/\left( R_{1660}+R_{680} \right)$ | [12] |
| Normalized difference between dRE and dG | EGFN=$\left( \max\left( D_{650:750} \right)-max\left( D_{500:550} \right) \right)/\left( \max\left( D_{650:750} \right)+max\left( D_{500:550} \right) \right)$ | [13] |
| Ratio of dRE and dG | EGFR=$\max\left( D_{650:750} \right)/max\left( D_{500:550} \right)$ | [13] |
| Enhanced vegetation index | EVI=$2.5\cdot\left( \left( R_{800}-R_{670} \right)/ \right.\left. \left( R_{800}-\left( 6\cdot R_{670} \right)-\left( 7.5\cdot R_{475} \right)+1 \right) \right)$ | [14] |
| Generalized difference vegetation index | GDVI=$\left( R_{800}^{n}-R_{680}^{n} \right)/\left( R_{800}^{n}+R_{680}^{n} \right)^{**}$ | [15] |
| Greenness index | GI=$R_{554}/R_{677}$ | [16] |
| Gitelson | Gitelson=$1/R_{700}$ | [17] |
|  | Gitelson2=$\left( R_{750}-R_{800}/R_{695}-R_{740} \right)-1$ | [6] |
| Gitelson and merzylak index | GMI1=$R_{750}/R_{550}$ | [6] |
|  | GMI2=$R_{750}/R_{700}$ | [6] |
| Green normalized difference vegetation index | Green NDVI=$\left( R_{800}-R_{550} \right)/\left( R_{800}+R_{550} \right)$ | [18] |
| Leaf water vegetation indices | LWVI_1=$\left( R_{1094}-R_{983} \right)/\left( R_{1094}+R_{983} \right)$ | [19] |
|  | LWVI_2=$\left( R_{1094}-R_{1205} \right)/\left( R_{1094}+R_{1205} \right)$ | [19] |
| Maccioni | Maccioni=$\left( R_{780}-R_{710} \right)/\left( R_{780}-R_{680} \right)$ | [20] |
| Modified chlorophyll absorption ratio index | MCARI=$\left( \left( R_{700}-R_{670} \right)-0.2\cdot\left( R_{700}-R_{550} \right) \right).\left( R_{700}/R_{670} \right)$ | [21] |
|  | MCARI2=$\left( \left( R_{750}-R_{705} \right)-0.2\cdot\left( R_{750}-R_{550} \right) \right).\left( R_{750}/R_{705} \right)$ | [22] |
| Combined MCARI/OSAVI | MCARI/OSAVI | [21] |
|  | MCARI2/OSAVI2 | [22] |
| Modified NDVI | mND705=$\left( R_{750}-R_{705} \right)/\left( R_{750}+R_{705}-2\cdot R_{445} \right)$ | [23] |
|  | mNDVI=$\left( R_{800}-R_{680} \right)/\left( R_{800}+R_{680}-2\cdot R_{445} \right)$ | [23] |
| Modified photochemical reflectance index | MPRI=$\left( R_{515}-R_{530} \right)/\left( R_{515}+R_{530} \right)$ | [24] |
| Modified red‐edge inflection point | mREIP= Red-edge inflection point using Gaussain fit | [25] |
| Modified soil adjusted vegetation index | MSAVI=$0.5\cdot\left( 2\cdot R_{800}+1-\left. \left( \left( 2\cdot R_{800}+1 \right)^{2}-8\cdot\left( R_{800}-R_{670} \right) \right)^{0.5} \right) \right.$ | [26] |
| Moisture stress index | MSI=$R_{1600}/R_{817}$ | [27] |
| Modified SR | mSR=$\left( R_{800}-R_{445} \right)/\left( R_{680}-R_{445} \right)$ | [23] |
|  | mSR2=$\left( R_{750}/R_{705} \right)-1/\left( R_{750}/R_{705}+1 \right)^{0.5}$ | [28] |
|  | mSR705=$\left( R_{750}-R_{445} \right)/\left( R_{705}-R_{445} \right)$ | [23] |
| Meris terrestrial chlorophyll index | MTCI=$\left( R_{754}-R_{709} \right)/\left( R_{709}-R_{681} \right)$ | [29] |
| Modified triangular vegetation index | MTVI=$1.2\cdot\left( 1.2\cdot\left( R_{800}-R_{550} \right)-\left. 2.5\cdot\left( R_{670}-R_{550} \right) \right) \right.$ | [30] |
| Normalized difference lignin index | NDLI=$\left( log\left( 1/R_{1754} \right)-log\left( 1/R_{1680} \right) \right)/\left( log\left( 1/R_{1754} \right)+log\left( 1/R_{1680} \right) \right)$ | [31] |
| Normalized difference nitrogen index | NDNI=$\left( log\left( 1/R_{1510} \right)-log\left( 1/R_{1680} \right) \right)/\left( log\left( 1/R_{1510} \right)+log\left( 1/R_{1680} \right) \right)$ | [31] |
| Normalized difference vegetation index | NDVI=$\left( R_{800}-R_{680} \right)/\left( R_{800}+R_{680} \right)$ | [32] |
|  | NDVI2=$\left( R_{750}-R_{705} \right)/\left( R_{750}+R_{705} \right)$ | [33] |
|  | NDVI3=$\left( R_{682}-R_{553} \right)/\left( R_{682}+R_{553} \right)$ | [34] |
| Normalized difference water index | NDWI=$\left( R_{860}-R_{1240} \right)/\left( R_{860}+R_{1240} \right)$ | [35] |
| Normalized pigments chlorophyll ratio index | NPCI=$\left( R_{680}-R_{430} \right)/\left( R_{680}+R_{430} \right)$ | [13] |
| Optimized soil-adjusted vegetation index | OSAVI=$(1+0.16)\cdot\left( R_{800}-R_{670} \right)/\left( R_{800}+R_{670}+0.16 \right)$ | [36] |
|  | OSAVI2=$(1+0.16)\cdot\left( R_{750}-R_{705} \right)/\left( R_{750}+R_{705}+0.16 \right)$ | [22] |
| Photochemical reflectance index | PRI=$\left( R_{531}-R_{570} \right)/\left( R_{531}+R_{570} \right)$ | [37] |
| Combined PRI/RDVI | PRI_norm=PRI $\cdot(-1)/\left( \right.$RDVI $\left. \cdot R_{700}/R_{670} \right)$ | [38] |
| Combined PRI/CI2 | PRI*CI2 | [39] |
| Plant senescence reflectance index | PSRI=$\left( R_{678}-R_{500})/R_{750} \right.$ | [40] |
| Pigment specific simple ratio | PSSR=$R_{800}/R_{635}$ | [41] |
| Pigment specific normalized difference | PSND=$\left( R_{800}-R_{470} \right)/\left( R_{800}-R_{470} \right)$ | [41] |
| Plant water index | PWI=$R_{900}/R_{970}$ | [42] |
| Renormalized difference  vegetation index | RDVI=$\left( R_{800}-R_{670} \right)/\sqrt{R_{800}+R_{670}}$ | [43] |
| Red-edge position | REP_LE= Red-edge position through linear extrapolation. | [44] |
|  | REP_Li=$R_{re}=\left( R_{670}+R_{780} \right)/2700+40\cdot\left( \left( R_{re}-R_{700} \right)/\left( R_{740}-R_{700} \right) \right)$ | [45] |
| Soil adjusted vegetation index | SAVI=$(1+L)\cdot\left( R_{800}-R_{670} \right)/\left( R_{800}+R_{670}+L \right)$ | [46] |
| Structure insensitive pigment index | SIPI=$\left( R_{800}-R_{445} \right)/\left( R_{800}-R_{680} \right)$ | [47] |
| Spectral polygon vegetation index | SPVI=$0.4\cdot3.7\cdot\left( R_{800}-R_{670} \right)-1.2\cdot\left( \left( R_{530}-R_{670} \right)^{2} \right)^{0.5}$ | [46] |
| Simple ratio | SR=$R_{800}/R_{680}$ | [48] |
|  | SR1=$R_{750}/R_{700}$ | [49] |
|  | SR2=$R_{752}/R_{690}$ | [49] |
|  | SR3=$R_{750}/R_{550}$ | [49] |
|  | SR4=$R_{700}/R_{670}$ | [50] |
|  | SR5=$R_{675}/R_{700}$ | [51] |
|  | SR6=$R_{750}/R_{710}$ | [52] |
|  | SR7=$R_{440}/R_{690}$ | [53] |
|  | SR8=$R_{515}/R_{550}$ | [54] |
|  | SR9=$R_{746}/R_{513}$ | [51] |
| Simple ratio pigment index | SRPI=$R_{430}/R_{680}$ | [55] |
| Simple ratio water index | SRWI=$R_{850}/R_{1240}$ | [5] |
| Area below the first derivative spectral curve | Sum_Dr1=$\sum_{i=626}^{795} D1_{i}$ | [56] |
|  | Sum_Dr2=$\sum_{i=680}^{780} D1_{i}$ | [57] |
| Short wave InfraRed fine particles index | SWIR FI=$R_{2133}^{2}/\left( R_{2225}\cdot R_{2209}^{3} \right.)$ | [58] |
| Short Wave InfraRed litter Index | SWIR LI=$3.87\cdot\left( R_{2210}-R_{2090} \right)-27.51\cdot\left( R_{2280}-R_{2090} \right)-0.2$ | [59] |
| Short wave InfraRed soil index | SWIR SI=$-41.59\cdot\left( R_{2210}-R_{2090} \right)+1.24\cdot\left( R_{2280}-R_{2090} \right)+0.64$ | [59] |
| Short wave InfraRed vegetation Index | SWIR VI=$37.72\cdot\left( R_{2210}-R_{2090} \right)+26.27\cdot\left( R_{2280}-R_{2090} \right)+0.57$ | [59] |
| Transformed Chlorophyll  absorption in reflectance index | TCARI=$3\cdot\left( \left( R_{700}-R_{670} \right)-0.2\cdot\left( R_{700}-R_{550} \right) \right.\cdot\left. \left( R_{700}/R_{670} \right) \right)$ | [30] |
|  | TCARI2=$3\cdot\left( \left( R_{750}-R_{705} \right)-0.2\cdot\left( R_{750}-R_{550} \right)\cdot\right.\left. \left( R_{750}/R_{705} \right) \right)$ | [22] |
| Combined TCARI/OSAVI | TCARI/OSAVI | [30] |
|  | TCARI2/OSAVI2 | [22] |
| Triangular greenness index | TGI=$-0.5\left( 190\left( R_{670}-R_{550} \right)-120\left( R_{670}-R_{480} \right) \right)$ | [60] |
| Triangular vegetation Index | TVI=$0.5\cdot\left( 120\cdot\left( R_{750}-R_{550} \right)-\left. 200\cdot\left( R_{670}-R_{550} \right) \right) \right.$ | [61] |
| Vogelmann | Vogelmann=$R_{740}/R_{720}$ | [62] |
|  | Vogelmann2=$\left( R_{734}-R_{747} \right)/\left( R_{715}+R_{726} \right)$ | [62] |
|  | Vogelmann3=$D_{715}/D_{705}$ | [62] |
|  | Vogelmann4=$\left( R_{734}-R_{747} \right)/\left( R_{715}+R_{720} \right)$ | [62] |

**Table S2** Vegetation indices ranking of each feature selection method at flowering.

| **Rank** | **MDI** | **Boruta** | **FeaLect** | **Relief** |
| --- | --- | --- | --- | --- |
| 1 | **PRI_norm** | **PRI_norm** | **REP_Li** | **REP_Li** |
| 2 | **Datt7** | **Datt7** | **Datt3** | **mREIP** |
| 3 | **Datt8** | **Datt8** | **PRI_norm** | **GDVI_4** |
| 4 | **REP_Li** | **PRI** | **Datt8** | **PRI_norm** |
| 5 | **PRI** | **REP_Li** | **Datt7** | **PRI** |
| 6 | **Datt3** | **Datt3** | **PRI** | **SWIR_VI** |
| 7 | **DPI** | **PRI_ch_CI2** | **Vogelmann2** | **OSAVI2** |
| 8 | **NDNI** | **Vogelmann2** | **Vogelmann4** | **MSAVI** |
| 9 | **D1** | **mREIP** | **Datt2** | **MCARI2** |
| 10 | **PWI** | **DPI** | **Datt** | **DPI** |
| 11 | **mREIP** | **Vogelmann4** | **DPI** | **GDVI_3** |
| 12 | **Vogelmann2** | **Datt** | **PRI_ch_CI2** | **OSAVI** |
| 13 | **REP_LE** | **PWI** | **mREIP** | **MCARI2_OSAVI2** |
| 14 | **Vogelmann4** | **Maccioni** | **NDNI** | **PSND** |
| 15 | **Carter** | **NDNI** | **Maccioni** | **SWIR_SI** |
| 16 | **PSRI** | **Datt2** | **MCARI2_OSAVI2** | **SR8** |
| 17 | **SR7** | **SRWI** | **TCARI2** | **SWIR_LI** |
| 18 | **Datt** | **D1** | **CAI** | **Datt7** |
| 19 | **SR8** | **NDWI** | **SWIR_VI** | **NDWI** |
| 20 | **PRI_ch_CI2** | **MTCI** | **PWI** | **TCARI2** |
| 21 | **LWVI2** | **SWIR_VI** | **LWVI1** | **DD** |
| 22 | **SWIR_VI** | **LWVI2** | **Carter4** | **DDn** |
| 23 | **EGFN** | **NPCI** | **NDLI** | **LWVI2** |
| 24 | **TGI** | **SRPI** | **SWIR_SI** | **PWI** |
| 25 | **mND705** | **Carter4** | **Vogelmann3** | **RDVI** |
| 26 | **SWIR_SI** | **TCARI2** | **SR7** | **mSR** |
| 27 | **DWSI2** | **SWIR_SI** | **D1** | **CRI2** |
| 28 | **EGFR** | **TGI** | **MCARI2** | **SRWI** |
| 29 | **SRWI** | **LWVI1** | **PSRI** | **Datt8** |
| 30 | **LWVI1** | **REP_LE** | **SWIR_LI** | **SPVI** |
| 31 | **Datt4** | **MCARI2_OSAVI2** | **REP_LE** | **CAI** |
| 32 | **TCARI** | **SPVI** | **Carter** | **NDVI3** |
| 33 | **mSR705** | **MCARI2** | **TCARI2_OSAVI2** | **MTVI** |
| 34 | **mNDVI** | **SR8** | **mSR** | **NDLI** |
| 35 | **SRPI** | **SR7** | **DWSI2** | **GDVI_2** |
| 36 | **Datt2** | **Carter** | **SRPI** | **PSRI** |
| 37 | **SIPI** | **PSRI** | **SR8** | **MCARI_OSAVI** |
| 38 | **DWSI3** | Sum_Dr2 | **NPCI** | **Datt3** |
| 39 | **MCARI_OSAVI** | DD | **SWIR_FI** | **Vogelmann2** |
| 40 | **NDWI** | TCARI2_OSAVI2 | **TGI** | Vogelmann4 |
| 41 | **Vogelmann3** | SIPI | **EGFR** | Datt2 |
| 42 | **MTCI** | Sum_Dr1 | **LWVI2** | Datt |
| 43 | **Boochs2** | SAVI | **EGFN** | Carter4 |
| 44 | **CRI4** | MTVI | **PSND** | TCARI2_OSAVI2 |
| 45 | **MSI** | MSAVI | **D2** | Maccioni |
| 46 | **TCARI2** | mNDVI | **SRWI** | SR6 |
| 47 | **NDVI3** | RDVI | **CRI2** | MTCI |
| 48 | **NDLI** | OSAVI | **NDWI** | CI2 |
| 49 | **D2** | Vogelmann3 | **NDVI3** | Vogelmann |
| 50 | NPCI | Vogelmann | **CI** | Green_NDVI |
| 51 | MCARI2_OSAVI2 | Gitelson2 | SIPI | Gitelson2 |
| 52 | ClAInt | NDVI3 | DWSI3 | mSR2 |
| 53 | DDn | CI2 | SR6 | NDVI2 |
| 54 | TCARI_OSAVI | EGFN | mNDVI | Carter2 |
| 55 | CAI | OSAVI2 | mSR705 | GMI2 |
| 56 | MPRI | MCARI_OSAVI | MTCI | SR1 |
| 57 | CI | DDn | Green_NDVI | NDVI |
| 58 | MCARI2 | DWSI4 | mND705 | SR |
| 59 | SR3 | mSR705 | Carter5 | PSSR |
| 60 | MCARI | mND705 | MSI | GMI1 |
| 61 | CRI2 | EGFR | Datt4 | SR3 |
| 62 | Gitelson2 | MCARI | DD | SR9 |
| 63 | CRI1 | SR6 | CI2 | Carter3 |
| 64 | Maccioni | GI | MSAVI | SR2 |
| 65 | CRI3 | TCARI | MCARI_OSAVI | REP_LE |
| 66 | TCARI2_OSAVI2 | Datt4 | DDn | LWVI1 |
| 67 | Vogelmann | DWSI3 | DWSI4 | EGFN |
| 68 | DWSI4 | Carter2 | DWSI1 | EGFR |
| 69 | Green_NDVI | PSND | MPRI | MSI |
| 70 | DD | MSI | MCARI | D2 |
| 71 | SWIR_FI | EVI | GI | DWSI5 |
| 72 | MTVI | DWSI1 | Datt5 | mND705 |
| 73 | OSAVI | Green_NDVI | DWSI5 | mSR705 |
| 74 | SPVI | Boochs2 | Boochs2 | DWSI1 |
| 75 | Carter6 | mSR2 | SR5 | SAVI |
| 76 | RDVI | CARI | RDVI | D1 |
| 77 | Sum_Dr2 | NDVI2 | MTVI | Sum_Dr2 |
| 78 | Gitelson | SWIR_FI | CRI1 | Sum_Dr1 |
| 79 | MSAVI | Datt5 | OSAVI | Datt6 |
| 80 | GMI1 | Carter6 | TCARI | MPRI |
| 81 | Carter3 | SR1 | Vogelmann | DWSI3 |
| 82 | DWSI5 | GMI2 | Carter3 | Vogelmann3 |
| 83 | SAVI | TCARI_OSAVI | Carter2 | GI |
| 84 | SR4 | Carter5 | SR3 | DWSI4 |
| 85 | Carter4 | DWSI5 | Gitelson2 | CRI1 |
| 86 | Carter2 | Gitelson | Sum_Dr2 | Datt5 |
| 87 | PSND | DWSI2 | EVI | NDNI |
| 88 | EVI | TVI | CRI3 | TVI |
| 89 | CARI | GMI1 | SAVI | EVI |
| 90 | DWSI1 | PSSR | Gitelson | CI |
| 91 | GI | Carter3 | OSAVI2 | SR5 |
| 92 | SR2 | NDLI | CRI4 | SR4 |
| 93 | GDVI_4 | CI | GMI1 | TCARI_OSAVI |
| 94 | PSSR | Boochs | Datt6 | Carter |
| 95 | CI2 | SR3 | SPVI | DWSI2 |
| 96 | Sum_Dr1 | D2 | CARI | Gitelson |
| 97 | Carter5 | GDVI_2 | TCARI_OSAVI | Carter5 |
| 98 | OSAVI2 | GDVI_3 | SR9 | SR7 |
| 99 | mSR | SR2 | SR4 | CRI3 |
| 100 | SR9 | ClAInt | Carter6 | NPCI |
| 101 | SR5 | GDVI_4 | mSR2 | SRPI |
| 102 | Datt6 | SR | PSSR | Carter6 |
| 103 | SR6 | SR5 | NDVI2 | CRI4 |
| 104 | TVI | SR4 | ClAInt | Boochs2 |
| 105 | Boochs | NDVI | Sum_Dr1 | ClAInt |
| 106 | Datt5 | Datt6 | NDVI | CARI |
| 107 | GMI2 | CRI4 | Boochs | SWIR_FI |
| 108 | NDVI | CRI3 | GMI2 | TCARI |
| 109 | SR1 | SR9 | SR1 | Datt4 |
| 110 | NDVI2 | mSR | TVI | Boochs |
| 111 | mSR2 | CAI | SR2 | TGI |
| 112 | GDVI_2 | SWIR_LI | GDVI_4 | mNDVI |
| 113 | SR | MPRI | GDVI_3 | SIPI |
| 114 | GDVI_3 | CRI2 | SR | MCARI |
| 115 | SWIR_LI | CRI1 | GDVI_2 | PRI_ch_CI2 |

The selected features of each feature selection method are marked in bold.

**Table S3** Vegetation indices ranking of each feature selection method at early grain filling.

| **Rank** | **MDI** | **Boruta** | **FeaLect** | **Relief** |
| --- | --- | --- | --- | --- |
| 1 | **PWI** | **PWI** | **PWI** | **REP_Li** |
| 2 | **SRWI** | **SRWI** | **REP_Li** | **PWI** |
| 3 | **NDWI** | **NDWI** | **NDWI** | **NDVI** |
| 4 | **REP_Li** | **REP_Li** | **SRWI** | **GDVI_2** |
| 5 | **Datt8** | **Datt3** | **Datt3** | **CI** |
| 6 | **CI** | **LWVI2** | **Carter2** | **Carter4** |
| 7 | **Datt7** | **Datt8** | **CI2** | **Carter2** |
| 8 | **Datt3** | **LWVI1** | **Gitelson2** | **GDVI_3** |
| 9 | **D2** | **Datt7** | **LWVI2** | **OSAVI** |
| 10 | **EGFN** | **Carter4** | **Carter4** | **LWVI1** |
| 11 | **EGFR** | **CI** | **LWVI1** | **NDVI3** |
| 12 | **Carter4** | **MSI** | **Carter3** | **PSND** |
| 13 | **LWVI1** | **DWSI5** | **Datt7** | **NDNI** |
| 14 | **D1** | **DWSI4** | **Datt8** | **LWVI2** |
| 15 | **NDLI** | **NDVI3** | **CI** | **NDWI** |
| 16 | **NDNI** | **CI2** | **D2** | **Datt5** |
| 17 | **Vogelmann3** | **DWSI1** | **CAI** | **SR** |
| 18 | **DWSI2** | **Carter2** | **Vogelmann3** | **MSI** |
| 19 | **MSI** | **GI** | **DWSI2** | **GDVI_4** |
| 20 | **DWSI5** | **DWSI2** | **Datt2** | **NDLI** |
| 21 | **DWSI3** | **D2** | **DWSI5** | **SIPI** |
| 22 | **NDVI3** | **Datt5** | **GDVI_2** | **PSRI** |
| 23 | **DWSI1** | **EGFN** | **NDNI** | **Vogelmann3** |
| 24 | **CI2** | **EGFR** | **MSI** | **Datt8** |
| 25 | **PRI_norm** | **GDVI_2** | **DWSI4** | **CI2** |
| 26 | **Carter5** | **GDVI_4** | **EGFN** | **Green_NDVI** |
| 27 | **SR9** | **SR** | **DPI** | **PRI_norm** |
| 28 | **SR4** | **GDVI_3** | **NDVI3** | **CAI** |
| 29 | **GI** | **Datt2** | **SWIR_LI** | **mREIP** |
| 30 | **Boochs2** | **NDVI** | **EGFR** | **MPRI** |
| 31 | **REP_LE** | **NDLI** | **NDLI** | **Carter3** |
| 32 | DWSI4 | **Carter3** | **GI** | **DWSI3** |
| 33 | Carter2 | **REP_LE** | **SWIR_VI** | **DPI** |
| 34 | PRI_ch_CI2 | **Gitelson2** | **DWSI3** | **mSR** |
| 35 | PRI | **Green_NDVI** | **REP_LE** | **mNDVI** |
| 36 | mREIP | **Vogelmann2** | **SR** | **Datt7** |
| 37 | MPRI | **Datt** | PSRI | **SWIR_LI** |
| 38 | Datt5 | **Vogelmann4** | DWSI1 | **OSAVI2** |
| 39 | PSRI | **SR4** | Datt5 | **Datt4** |
| 40 | LWVI2 | **PSRI** | D1 | **SR8** |
| 41 | Datt4 | **SR5** | NDVI | **SWIR_FI** |
| 42 | SR5 | **D1** | SR8 | **SRWI** |
| 43 | SR8 | **PRI_norm** | PRI | Datt3 |
| 44 | Datt2 | **DWSI3** | SWIR_SI | Gitelson2 |
| 45 | SWIR_FI | **SR6** | Green_NDVI | DWSI5 |
| 46 | Carter3 | **NDNI** | MPRI | SR2 |
| 47 | Vogelmann | **NDVI2** | Datt | PSSR |
| 48 | PSSR | **TCARI2_OSAVI2** | TCARI2_OSAVI2 | Datt2 |
| 49 | PSND | **Carter5** | PRI_ch_CI2 | GMI2 |
| 50 | CRI2 | **PRI** | GDVI_3 | SR1 |
| 51 | Vogelmann2 | **GMI2** | Carter5 | mSR2 |
| 52 | SWIR_SI | **SR1** | PRI_norm | NDVI2 |
| 53 | mNDVI | **mSR2** | Vogelmann2 | TCARI2_OSAVI2 |
| 54 | MTCI | **SR2** | MCARI_OSAVI | SR6 |
| 55 | EVI | **Vogelmann3** | SWIR_FI | Vogelmann2 |
| 56 | Green_NDVI | **PRI_ch_CI2** | Boochs2 | TCARI2 |
| 57 | Datt | **TCARI2** | Vogelmann4 | Vogelmann4 |
| 58 | TCARI2_OSAVI2 | **Maccioni** | CRI2 | DWSI1 |
| 59 | SIPI | **PSND** | SR4 | GMI1 |
| 60 | MCARI_OSAVI | **TGI** | TCARI2 | SR3 |
| 61 | mSR705 | **PSSR** | SR9 | Vogelmann |
| 62 | TGI | **OSAVI** | GDVI_4 | Maccioni |
| 63 | Gitelson | **Boochs2** | SR5 | DWSI4 |
| 64 | Gitelson2 | **SIPI** | Carter | SR9 |
| 65 | GDVI_2 | Vogelmann | SR2 | GI |
| 66 | NPCI | mND705 | SIPI | mND705 |
| 67 | Vogelmann4 | MTCI | mSR | mSR705 |
| 68 | Carter | MCARI2 | mREIP | Datt |
| 69 | mSR | mNDVI | PSSR | MTCI |
| 70 | DPI | mSR705 | PSND | REP_LE |
| 71 | DDn | MCARI2_OSAVI2 | TGI | MCARI2_OSAVI2 |
| 72 | CRI3 | MPRI | Maccioni | MCARI2 |
| 73 | SRPI | SR9 | MCARI | PRI_ch_CI2 |
| 74 | Sum_Dr1 | OSAVI2 | Vogelmann | D1 |
| 75 | TCARI | GMI1 | SR7 | PRI |
| 76 | Boochs | Datt4 | GMI2 | Datt6 |
| 77 | GMI1 | SR3 | GMI1 | SR5 |
| 78 | CARI | DDn | CRI1 | SR4 |
| 79 | mND705 | TCARI | mNDVI | EGFN |
| 80 | SR2 | MCARI_OSAVI | Datt4 | EGFR |
| 81 | ClAInt | MSAVI | mSR2 | Carter5 |
| 82 | GDVI_4 | SWIR_FI | SR6 | CRI1 |
| 83 | GDVI_3 | TCARI_OSAVI | NPCI | MSAVI |
| 84 | MCARI | SAVI | OSAVI2 | D2 |
| 85 | SR3 | mSR | SRPI | RDVI |
| 86 | Datt6 | SR8 | NDVI2 | DD |
| 87 | NDVI2 | MTVI | MCARI2_OSAVI2 | SAVI |
| 88 | Maccioni | MCARI | Datt6 | Gitelson |
| 89 | SWIR_VI | RDVI | Boochs | NPCI |
| 90 | MCARI2 | Sum_Dr2 | mND705 | SRPI |
| 91 | CRI4 | SPVI | OSAVI | SPVI |
| 92 | TCARI2 | Sum_Dr1 | MCARI2 | Sum_Dr2 |
| 93 | MTVI | Boochs | SR3 | DDn |
| 94 | SR6 | Datt6 | mSR705 | MTVI |
| 95 | SR | mREIP | DDn | Sum_Dr1 |
| 96 | mSR2 | DD | TCARI | SWIR_SI |
| 97 | NDVI | Gitelson | TCARI_OSAVI | ClAInt |
| 98 | GMI2 | Carter6 | SR1 | CRI2 |
| 99 | CRI1 | CARI | CRI4 | Carter6 |
| 100 | TCARI_OSAVI | ClAInt | Carter6 | TCARI_OSAVI |
| 101 | DD | TVI | ClAInt | CRI3 |
| 102 | OSAVI | SWIR_SI | Gitelson | SWIR_VI |
| 103 | SR7 | CRI2 | MTCI | CRI4 |
| 104 | MCARI2_OSAVI2 | SRPI | DD | TVI |
| 105 | TVI | EVI | CRI3 | CARI |
| 106 | OSAVI2 | NPCI | EVI | SR7 |
| 107 | SR1 | CRI1 | CARI | EVI |
| 108 | MSAVI | SR7 | TVI | Boochs |
| 109 | SPVI | DPI | MSAVI | TCARI |
| 110 | Carter6 | CRI3 | MTVI | Carter |
| 111 | SAVI | CRI4 | SPVI | MCARI |
| 112 | CAI | Carter | Sum_Dr1 | TGI |
| 113 | RDVI | CAI | RDVI | MCARI_OSAVI |
| 114 | Sum_Dr2 | SWIR_VI | SAVI | Boochs2 |
| 115 | SWIR_LI | SWIR_LI | Sum_Dr2 | DWSI2 |

The selected features of each feature selection method are marked in bold.

**Table S4** Vegetation indices ranking of each feature selection method at mid grain filling.

| **Rank** | **MDI** | **Boruta** | **FeaLect** | **Relief** |
| --- | --- | --- | --- | --- |
| 1 | **PWI** | **LWVI2** | **LWVI2** | **LWVI2** |
| 2 | **LWVI1** | **LWVI1** | **Datt3** | **REP_Li** |
| 3 | **LWVI2** | **PWI** | **NDWI** | **LWVI1** |
| 4 | **Datt3** | **Datt3** | **LWVI1** | **Vogelmann3** |
| 5 | **REP_Li** | **SRWI** | **SRWI** | **GDVI_3** |
| 6 | **NDWI** | **NDWI** | **PWI** | **GDVI_2** |
| 7 | **SRWI** | **REP_Li** | **REP_Li** | **GDVI_4** |
| 8 | **DWSI1** | **Vogelmann2** | **Carter4** | **OSAVI** |
| 9 | **MSI** | **DWSI5** | **Vogelmann4** | **OSAVI2** |
| 10 | **NDLI** | **Vogelmann4** | **Vogelmann2** | **PWI** |
| 11 | **Vogelmann3** | **Carter4** | **DWSI1** | **Carter4** |
| 12 | **DWSI5** | **MSI** | **DWSI5** | **DD** |
| 13 | **CI** | **DWSI1** | **MSI** | **DDn** |
| 14 | **DWSI2** | **Datt2** | **Green_NDVI** | **Carter3** |
| 15 | **MCARI_OSAVI** | **Carter3** | **SWIR_VI** | **DPI** |
| 16 | **Carter5** | **Gitelson2** | **SWIR_LI** | **CAI** |
| 17 | **NDNI** | **Green_NDVI** | **NDNI** | **NDWI** |
| 18 | **Datt5** | **PSSR** | **Vogelmann3** | **SAVI** |
| 19 | **mREIP** | **GDVI_4** | **CI** | **MSAVI** |
| 20 | **EGFN** | **CI2** | **DWSI4** | **D2** |
| 21 | **PRI_ch_CI2** | **NDVI** | **CAI** | **SR5** |
| 22 | **DWSI3** | **GDVI_3** | **Datt8** | **Datt3** |
| 23 | **MCARI** | **SR** | **NDLI** | **mSR** |
| 24 | **Vogelmann2** | **Datt** | **Datt5** | **RDVI** |
| 25 | **REP_LE** | **GDVI_2** | **D2** | **Datt5** |
| 26 | **D1** | **Vogelmann** | **DPI** | **EVI** |
| 27 | **OSAVI** | **Carter2** | **PSSR** | **Green_NDVI** |
| 28 | **DWSI4** | **Datt5** | **Carter3** | **CI** |
| 29 | **CRI2** | **SR1** | **NDVI3** | **NDNI** |
| 30 | **D2** | **GMI1** | **SWIR_SI** | **PSND** |
| 31 | **PRI** | **SR3** | **DWSI2** | **SRWI** |
| 32 | **SR8** | **EGFN** | **GDVI_3** | **SR8** |
| 33 | Datt6 | **NDVI3** | **GDVI_4** | **MCARI2** |
| 34 | Datt4 | **D2** | **SR** | **NDLI** |
| 35 | CRI1 | **GI** | **NDVI** | **PSRI** |
| 36 | Carter4 | **Maccioni** | **Datt7** | **mNDVI** |
| 37 | GI | **GMI2** | **GI** | **MPRI** |
| 38 | SR4 | **TCARI2_OSAVI2** | **EGFN** | **mREIP** |
| 39 | SWIR_FI | **DWSI4** | **EGFR** | **Carter** |
| 40 | MTVI | **Carter5** | **SWIR_FI** | **Datt7** |
| 41 | TCARI_OSAVI | **Datt8** | **SR5** | **REP_LE** |
| 42 | DDn | **SWIR_VI** | **SR8** | **SWIR_SI** |
| 43 | EGFR | **Vogelmann3** | **OSAVI** | DWSI5 |
| 44 | OSAVI2 | **CI** | **REP_LE** | Vogelmann2 |
| 45 | SWIR_VI | **SR4** | **Carter5** | DWSI1 |
| 46 | mSR | **SWIR_LI** | MCARI_OSAVI | Vogelmann4 |
| 47 | NDVI3 | **SR6** | DWSI3 | MSI |
| 48 | DD | **SR5** | MCARI2 | Datt2 |
| 49 | Carter | **MTCI** | GDVI_2 | PSSR |
| 50 | PSSR | **DPI** | Datt2 | NDVI |
| 51 | PRI_norm | **DWSI2** | MCARI2_OSAVI2 | SR |
| 52 | Datt8 | **NDLI** | D1 | SR6 |
| 53 | EVI | **REP_LE** | OSAVI2 | CI2 |
| 54 | TCARI2_OSAVI2 | **EGFR** | SR4 | Vogelmann |
| 55 | SR5 | **OSAVI2** | MCARI | Carter2 |
| 56 | Vogelmann4 | **Datt7** | mREIP | Gitelson2 |
| 57 | Boochs2 | **NDNI** | CRI2 | TCARI2_OSAVI2 |
| 58 | Datt7 | **mSR2** | PRI_norm | Maccioni |
| 59 | Boochs | **CAI** | PRI | mSR2 |
| 60 | Carter3 | **SWIR_SI** | PSRI | NDVI2 |
| 61 | SR7 | **PSND** | PSND | TCARI2 |
| 62 | PSND | **DD** | CRI1 | Datt |
| 63 | mNDVI | **MCARI_OSAVI** | Carter2 | SR2 |
| 64 | Sum_Dr1 | **MCARI2** | Datt | GMI2 |
| 65 | mSR705 | **D1** | MPRI | SR1 |
| 66 | SIPI | **OSAVI** | PRI_ch_CI2 | GMI1 |
| 67 | MTCI | **DWSI3** | Maccioni | SR3 |
| 68 | TGI | **SR8** | Carter | MTCI |
| 69 | GDVI_2 | **TCARI2** | Vogelmann | EGFN |
| 70 | MSAVI | **SR2** | DD | EGFR |
| 71 | SR3 | **MCARI2_OSAVI2** | TCARI_OSAVI | MCARI2_OSAVI2 |
| 72 | MCARI2 | **mREIP** | SIPI | D1 |
| 73 | mND705 | **MCARI** | Datt4 | mND705 |
| 74 | MPRI | **PRI_norm** | SR9 | mSR705 |
| 75 | SR | **MPRI** | Boochs2 | SR9 |
| 76 | Gitelson2 | **Datt4** | GMI1 | SIPI |
| 77 | RDVI | **SWIR_FI** | SR3 | PRI_norm |
| 78 | GDVI_3 | **PSRI** | mSR | Datt6 |
| 79 | SWIR_LI | **RDVI** | CRI4 | PRI |
| 80 | CRI3 | **NDVI2** | TGI | DWSI4 |
| 81 | SR9 | **SAVI** | CRI3 | GI |
| 82 | CAI | **PRI** | DDn | NDVI3 |
| 83 | DPI | **PRI_ch_CI2** | TCARI | PRI_ch_CI2 |
| 84 | PSRI | TCARI | EVI | SPVI |
| 85 | Datt | CRI1 | CI2 | DWSI3 |
| 86 | SPVI | SPVI | CARI | Sum_Dr2 |
| 87 | GMI1 | mSR | mNDVI | NPCI |
| 88 | Green_NDVI | MTVI | SR2 | SRPI |
| 89 | Vogelmann | mNDVI | SRPI | Sum_Dr1 |
| 90 | TVI | CARI | NPCI | TCARI_OSAVI |
| 91 | Maccioni | CRI2 | RDVI | MTVI |
| 92 | NDVI | DDn | Gitelson2 | SR4 |
| 93 | GDVI_4 | Sum_Dr1 | MSAVI | SWIR_FI |
| 94 | CRI4 | SR9 | TCARI2_OSAVI2 | Gitelson |
| 95 | Datt2 | Boochs2 | SR7 | CRI1 |
| 96 | SAVI | CRI4 | MTCI | Carter5 |
| 97 | CI2 | CRI3 | Boochs | Carter6 |
| 98 | Carter2 | Carter | Datt6 | ClAInt |
| 99 | TCARI | SIPI | Carter6 | TVI |
| 100 | CARI | mND705 | Gitelson | SR7 |
| 101 | Carter6 | MSAVI | SR1 | CRI2 |
| 102 | MCARI2_OSAVI2 | TCARI_OSAVI | mSR705 | CARI |
| 103 | Gitelson | NPCI | SAVI | CRI4 |
| 104 | SRPI | Sum_Dr2 | SPVI | CRI3 |
| 105 | SR2 | Carter6 | mND705 | MCARI_OSAVI |
| 106 | NPCI | TGI | ClAInt | DWSI2 |
| 107 | Sum_Dr2 | EVI | TCARI2 | TGI |
| 108 | ClAInt | SR7 | Sum_Dr2 | TCARI |
| 109 | SWIR_SI | Gitelson | MTVI | Boochs |
| 110 | SR1 | mSR705 | Sum_Dr1 | Datt8 |
| 111 | TCARI2 | Boochs | TVI | SWIR_VI |
| 112 | mSR2 | TVI | GMI2 | Datt4 |
| 113 | NDVI2 | SRPI | mSR2 | SWIR_LI |
| 114 | GMI2 | Datt6 | SR6 | Boochs2 |
| 115 | SR6 | ClAInt | NDVI2 | MCARI |

The selected features of each feature selection method are marked in bold.

**Table S5** Vegetation indices ranking of each feature selection method at mid grain filling.

| **Rank** | **MDI** | **Boruta** | **FeaLect** | **Relief** |
| --- | --- | --- | --- | --- |
| 1 | **DPI** | **DPI** | **D1** | **LWVI2** |
| 2 | **NDNI** | **LWVI2** | **Vogelmann4** | **REP_Li** |
| 3 | **LWVI2** | **REP_LE** | **Vogelmann2** | **Datt** |
| 4 | **PRI_ch_CI2** | **SRWI** | **DPI** | **DPI** |
| 5 | **Vogelmann3** | **DWSI1** | **LWVI2** | **MCARI_OSAVI** |
| 6 | **REP_LE** | **NDWI** | **Datt2** | **D1** |
| 7 | **D1** | **NDNI** | **REP_Li** | **Datt3** |
| 8 | **LWVI1** | **Vogelmann3** | **REP_LE** | **mREIP** |
| 9 | **NDLI** | **DWSI5** | **NDWI** | **NDNI** |
| 10 | **DWSI1** | **D1** | **D2** | **SWIR_LI** |
| 11 | **DWSI5** | **PRI_ch_CI2** | **SRWI** | **REP_LE** |
| 12 | **PRI** | **PWI** | **NDNI** | **SWIR_VI** |
| 13 | **Datt8** | **PRI** | **Datt3** | **SR8** |
| 14 | **CI** | **LWVI1** | **MSI** | **Datt8** |
| 15 | **NDWI** | **SWIR_FI** | **Vogelmann** | **EGFN** |
| 16 | **PWI** | **Datt8** | **Maccioni** | **PRI** |
| 17 | **SRWI** | **MSI** | **DWSI1** | **SWIR_FI** |
| 18 | **SR8** | **Datt3** | **PRI_ch_CI2** | **CAI** |
| 19 | **SWIR_VI** | **Vogelmann2** | **LWVI1** | **NDWI** |
| 20 | **SWIR_FI** | **D2** | **Vogelmann3** | **DWSI2** |
| 21 | **D2** | **Maccioni** | **NDLI** | **PWI** |
| 22 | **Carter** | **NDLI** | **PWI** | **Carter** |
| 23 | **SRPI** | **Vogelmann4** | **DD** | **SWIR_SI** |
| 24 | **DWSI3** | **CI** | **PRI** | **MCARI** |
| 25 | **SR5** | **Carter** | **Carter** | **D2** |
| 26 | **Datt5** | **REP_Li** | **DWSI5** | **NDLI** |
| 27 | **SWIR_LI** | **MTCI** | **Datt8** | **DDn** |
| 28 | **Boochs2** | **DWSI3** | **SWIR_LI** | **LWVI1** |
| 29 | Carter5 | **NDVI3** | **SWIR_VI** | **SRWI** |
| 30 | PRI_norm | **PRI_norm** | **CAI** | **MSI** |
| 31 | Vogelmann2 | **PSRI** | **SWIR_SI** | **Datt7** |
| 32 | MPRI | **GI** | **EGFR** | **Datt4** |
| 33 | NPCI | **Carter5** | **CI** | **EVI** |
| 34 | TGI | **NPCI** | **EGFN** | **TCARI_OSAVI** |
| 35 | REP_Li | **Boochs** | **Datt7** | **Green_NDVI** |
| 36 | Vogelmann4 | Datt5 | **SR8** | **ClAInt** |
| 37 | Boochs | DWSI4 | **SWIR_FI** | **Boochs** |
| 38 | PSRI | SR4 | **SRPI** | **CRI4** |
| 39 | NDVI3 | SRPI | **Boochs** | **TCARI2** |
| 40 | Gitelson | Vogelmann | **DWSI2** | **EGFR** |
| 41 | CRI1 | SR5 | **Datt** | **PRI_ch_CI2** |
| 42 | MSI | Datt2 | **DWSI3** | **TCARI2_OSAVI2** |
| 43 | SR4 | DD | **Datt5** | **TGI** |
| 44 | PSND | SR7 | **NPCI** | **Carter5** |
| 45 | ClAInt | Datt | **Boochs2** | **Vogelmann3** |
| 46 | GI | Boochs2 | **SR7** | **mSR** |
| 47 | CRI3 | SR8 | **NDVI3** | DWSI1 |
| 48 | EVI | mSR705 | **GI** | DWSI5 |
| 49 | Maccioni | mSR | **DDn** | Vogelmann2 |
| 50 | DWSI2 | OSAVI2 | PSND | Vogelmann4 |
| 51 | Datt3 | DWSI2 | Green_NDVI | Datt2 |
| 52 | TCARI | mND705 | MCARI | Vogelmann |
| 53 | Datt2 | SIPI | PRI_norm | Maccioni |
| 54 | Datt4 | ClAInt | PSRI | SR6 |
| 55 | SIPI | mNDVI | Carter5 | MTCI |
| 56 | SWIR_SI | SR6 | PSSR | mSR2 |
| 57 | CARI | TVI | DWSI4 | NDVI2 |
| 58 | RDVI | MCARI_OSAVI | MTCI | Carter4 |
| 59 | DWSI4 | mREIP | CRI2 | PSSR |
| 60 | CRI2 | Carter4 | MCARI_OSAVI | Gitelson2 |
| 61 | DD | PSND | EVI | DD |
| 62 | Sum_Dr2 | MCARI | GMI1 | GMI1 |
| 63 | mREIP | SWIR_LI | SR5 | SR3 |
| 64 | SR9 | TCARI2 | Datt4 | mND705 |
| 65 | MTVI | Green_NDVI | TGI | mSR705 |
| 66 | Sum_Dr1 | MCARI2_OSAVI2 | SR4 | GMI2 |
| 67 | mSR | MTVI | SR6 | SR1 |
| 68 | Carter6 | SWIR_VI | MPRI | GDVI_2 |
| 69 | TCARI_OSAVI | Gitelson | TCARI2_OSAVI2 | GDVI_3 |
| 70 | SR7 | NDVI2 | mREIP | GDVI_4 |
| 71 | Green_NDVI | SPVI | SR9 | NDVI |
| 72 | MCARI | EVI | CRI3 | SR |
| 73 | TCARI2 | CARI | TCARI | SR2 |
| 74 | MTCI | SR9 | SIPI | CI2 |
| 75 | SAVI | SAVI | CRI1 | OSAVI2 |
| 76 | MSAVI | MCARI2 | SR3 | Datt6 |
| 77 | mNDVI | Sum_Dr2 | CRI4 | Carter3 |
| 78 | TVI | OSAVI | OSAVI2 | Carter2 |
| 79 | OSAVI2 | Datt4 | CARI | mNDVI |
| 80 | Datt6 | mSR2 | TCARI_OSAVI | SIPI |
| 81 | MCARI2_OSAVI2 | SR3 | mSR | MCARI2 |
| 82 | mND705 | Sum_Dr1 | Datt6 | MCARI2_OSAVI2 |
| 83 | EGFR | Carter6 | ClAInt | PSRI |
| 84 | OSAVI | MPRI | Gitelson | PRI_norm |
| 85 | SR3 | MSAVI | Carter6 | OSAVI |
| 86 | MCARI_OSAVI | GMI1 | Carter3 | PSND |
| 87 | TCARI2_OSAVI2 | TCARI_OSAVI | Carter4 | SR9 |
| 88 | SPVI | RDVI | OSAVI | Gitelson |
| 89 | CRI4 | PSSR | MCARI2 | RDVI |
| 90 | Carter2 | TGI | Sum_Dr2 | CARI |
| 91 | GMI1 | SR1 | Sum_Dr1 | SAVI |
| 92 | mSR705 | TCARI | TVI | NPCI |
| 93 | SR2 | CRI3 | SPVI | SRPI |
| 94 | Datt7 | CRI4 | mNDVI | MSAVI |
| 95 | Datt | EGFR | TCARI2 | Datt5 |
| 96 | MCARI2 | EGFN | MCARI2_OSAVI2 | DWSI4 |
| 97 | Carter3 | CRI1 | Gitelson2 | NDVI3 |
| 98 | Vogelmann | SWIR_SI | MTVI | GI |
| 99 | DDn | GDVI_2 | SAVI | SR7 |
| 100 | Carter4 | Gitelson2 | mSR705 | Carter6 |
| 101 | GDVI_3 | CI2 | RDVI | MPRI |
| 102 | GDVI_4 | GDVI_4 | GMI2 | DWSI3 |
| 103 | EGFN | Carter3 | mND705 | CRI1 |
| 104 | mSR2 | NDVI | MSAVI | Sum_Dr2 |
| 105 | CI2 | TCARI2_OSAVI2 | SR2 | SPVI |
| 106 | Gitelson2 | GMI2 | NDVI2 | MTVI |
| 107 | SR6 | Carter2 | Carter2 | CRI3 |
| 108 | SR | SR2 | SR | Sum_Dr1 |
| 109 | PSSR | SR | CI2 | SR5 |
| 110 | CAI | Datt6 | GDVI_4 | SR4 |
| 111 | GMI2 | GDVI_3 | GDVI_3 | TVI |
| 112 | GDVI_2 | DDn | NDVI | Boochs2 |
| 113 | NDVI | CRI2 | GDVI_2 | CRI2 |
| 114 | SR1 | Datt7 | SR1 | CI |
| 115 | NDVI2 | CAI | mSR2 | TCARI |

The selected features of each feature selection method are marked in bold.

**References**

[1] Boochs F, Kupfer G, Dockter K, Ku¨hbauch W. Shape of the red edge as vitality indicator for plants. Int J Remote Sens. 1990;11 (10):1741–1753.

[2] Nagler PL, Inoue Y, Glenn E, Russ A, Daughtry C. Cellulose absorption index (CAI) to quantify mixed soil-plant litter scenes. Remote Sens Environ. 2003;87 (2-3):310–325.

[3] Kim M, Daughtry C, Chappelle E, McMurtrey J, Walthall C. The use of high spectral resolution bands for estimating absorbed photosynthetically active radiation. (Apar) In: Proceedings of the Sixth Symposium on Physical Measurements and Signatures in Remote Sensing Val D’Isere, France, 1994, pp 299–306

[4] Carter GA. Ratios of leaf reﬂectances in narrow wavebands as indicators of plant stress. Int J Remote Sens. 1994;15(3):697–703.

[5] Zarco-Tejada PJ, Pushnik JC, Dobrowski S, Ustin SL. Steadystate chlorophyll a ﬂuorescence detection from canopy derivative reﬂectance and double-peak red-edge eﬀects. Remote Sens Environ. 2003;84(2):283–294.

[6] Gitelson A, Y G, MN, M. Relationships between leaf chlorophyll content and spectral reﬂectance and algorithms for non-destructive chlorophyll assessment in higher plant leaves. Plant Physiol. 2003;160 (3):271–282.

[7] Datt B. Visible/near infrared reﬂectance and chlorophyll content in Eucalyptus leaves. Int J Remote Sens. 1999;20(14):2741–2759.

[8] Datt B. Remote sensing of chlorophyll a, chlorophyll b, chlorophyll a+b, and total carotenoid content in Eucalyptus leaves. Remote Sens Environ. 1998;66(2): 111–121.

[9] Datt B. Remote sensing of water content in Eucalyptus leaves. Aust J Bot. 1999;47(6):909–923.

[10] le Maire G, Francoi C, Dufrene E. Towards universal broad leaf chlorophyll indices using PROSPECT simulated database and hyperspectral reﬂectance measurements. Remote Sens Environ. 2004;89 (1):1–28.

[11] le Maire G, Fran¸cois C, Soudani K, Berveiller D, Pontailler J -Y, Br´eda N et al. Calibration and validation of hyperspectral indices for the estimation of broadleaved forest leaf chlorophyll content, leaf mass per area, leaf area index and leaf canopy biomass. Remote Sens Environ. 2008;112(10):3846–3864.

[12] Apan A, Held A, Phinn S, Markley J, Jan. Detecting sugarcane “orange rust” disease using EO-1 Hyperion hyperspectral imagery. Int J Remote Sens. 2004;25 (2):489–498.

[13] Pen˜uelas J, Gamon JA, Fredeen AL, Merino J, Field CB. Reﬂectance indexes associated with physiological-changes in nitrogen-limited and water-limited sunﬂower leaves Remote Sens Environ. 1994;48 (2):135–146.

[14] Huete A, Liu H, Batchily K, van Leeuwen W. A comparison of vegetation indices over a global set of TM images for EOS-MODIS. Remote Sens Environ. 1997;59 (3):440–451.

[15] Wu W, The generalized diﬀerence vegetation index (GDVI) for dryland characterization. Remote Sens. 2014;6(2):1211–1233.

[16] Smith R, Adams J, Stephens D, Hick P. Forecasting wheat yield in a mediterranean-type environment from the NOAA satellite. Aust J Agr Res. 1995;46 (1):113–125.

[17] Gitelson A, Buschmann C, Lichtenthaler H. The chlorophyll ﬂuorescence ratio F735/F700 as an accurate measure of the chlorophyll content in plants Experiments with autumn chestnut and maple leaves. Remote Sens Environ. 1999;69 (3):296–302.

[18] Gitelson AA, Kaufman YJ, Merzlyak, MN. Use of a green channel in remote sensing of global vegetation from EOS-MODIS. Remote Sens Environ. 1996;58(3):289–298.

[19] Galv˜ao LS, Formaggio AR, Tisot DA. Discrimination of sugarcane varieties in southeastern Brazil with EO-1 Hyperion data. Remote Sens Environ. 2005;94(4):523–534.

[20] Maccioni A, Agati G, Mazzinghi P. New vegetation indices for remote measurement of chlorophylls based on leaf directional reﬂectance spectra. J Photoch Photobio B. 2001;61(1-2):52–61.

[21] Daughtry C, Walthall C, Kim M, de Colstoun E, J M. Estimating corn leaf chlorophyll concentration from leaf and canopy reﬂectance. Remote Sens Environ. 2000;74(2):229–239.

[22] Wu C, Niu Z, Tang Q, Huang W. Estimating chlorophyll content from hyperspectral vegetation indices: Modeling and validation. Agric For Meteorol. 2008;148(8-9):1230–1241.

[23] Sims D, Gamon J. Relationships between leaf pigment content and spectral reﬂectance across a wide range of species, leaf structures and developmental stages. Remote Sens Environ. 2002;81(2):337–354.

[24] Hern´andez-Clemente R, Navarro-Cerrillo RM, Su´arez L, Morales F, Zarco-Tejada PJ. Assessing structural eﬀects on PRI for stress detection in conifer forests. Remote Sens Environ. 2011;115(9):2360–2375.

[25] Miller JR, Hare EW, Wu J. Quantitative characterisation of the red edge reflectance An inverted-Gaussian model. Int J Remote Sens. 1990;11:1755-1773.

[26] Qi J, Chehbouni A, Huete A, Kerr Y, Sorooshian S. A modiﬁed soil adjusted vegetation index. Remote Sens Environ. 1994;48(2):119–126.

[27] Hunt ER, Rock BN. Detection of changes in leaf water-content using near-infrared and middle-infrared reﬂectances. Remote Sens Environ. 1989;30 (1):43–54.

[28] Chen JM. Evaluation of vegetation indices and a modiﬁed simple ratio for boreal applications. Can J Remote Sens. 1996;22:229–242.

[29] Dash J, Curran PJ. The MERIS terrestrial chlorophyll index. Int J Remote Sens. 2004;25(23):5403–5413.

[30] Haboudane D, Miller JR, Tremblay N, Zarco-Tejada PJ, Dextraze L. Integrated narrow-band vegetation indices for prediction of crop chlorophyll content for application to precision agriculture. Remote Sens Environ. 2002;81(2-3), PII S0034–4257(02)00018–4.

[31] Serrano L, Pen˜uelas J, Ustin SL. Remote sensing of nitrogen and lignin in mediterranean vegetation from AVIRIS data: Decomposing biochemical from structural signals. Remote Sens Environ. 2002;81:355–364.

[32] Tucker CJ. Red and photographic infrared linear combinations for monitoring vegetation. Remote Sens Environ. 1979;8(2):127–150.

[33] Gitelson A, Merzlyak MN. Quantitative estimation of chlorophylla using reﬂectance spectra: Experiments with autumn chestnut and maple leaves. J Photoch Photobio B. 1994;22(3):247–252.

[34] Gandia S, Fern´andez G, Garc´ıa J, Moreno J. Retrieval of vegetation biophysical variables from CHRIS/PROBA data in the SPARC campaign In: ESA SP Vol 578 2004, pp 40–48.

[35] Gao B -c. NDWI A normalized diﬀerence water index for remote sensing of vegetation liquid water from space. Remote Sens Environ. 1996;58(3):257–266.

[36] Rondeaux G, Steven M, Baret F. Optimization of soil-adjusted vegetation indices. Remote Sens Environ. 1996;55(2):95–107.

[37] Gamon J, nuelas JP, Field C. A narrow-waveband spectral index that tracks diurnal changes in photosynthetic eﬃciency. Remote Sens Environ. 1992;41 (1):35–44.

[38] Zarco-Tejada PJ, Gonzalez-Dugo V, Williams LE, Suarez L, Berni JAJ, Goldhamer D, Fereres E. A PRI-based water stress index combining structural and chlorophyll eﬀects: Assessment using diurnal narrow-band airborne imagery and the CWSI thermal index. Remote Sens Environ. 2013;138:38–50.

[39] Garrity SR, Eitel JU, Vierling LA. Disentangling the relationships between plant pigments and the photochemical reﬂectance index reveals a new approach for remote estimation of carotenoid content. Remote Sens Environ. 2011;115 (2):628–635.

[40] Merzlyak MN, Gitelson AA, Chivkunova OB, Rakitin VY. Non-destructive optical detection of pigment changes during leaf senescence and fruit ripening. Physiol Plant. 1999;106(1):135–141.

[41] Blackburn GA., Quantifying chlorophylls and caroteniods at leaf and canopy scales: An evaluation of some hyperspectral approaches. Remote Sens Environ. 1998;66(3):273–285.

[42] Pen˜uelas J, Pin˜ol J, Ogaya R, Filella I. Estimation of plant water concentration by the reﬂectance water index WI (R900/R970). Int J Remote Sens. 1997;18(13):2869–2875.

[43] Roujean JL, Breon FM. Estimating par absorbed by vegetation from bidirectional reﬂectance measurements. Remote Sens Environ. 1995;51(3):375–384.

[44] Cho MA，Skidmore A K. A new technique for extracting the red edge position from hyperspectral data: The linear extrapolation method. Remote Sensing of Environment, 2006;101:181.

[45] Guyot G, Baret F. Utilisation de la haute resolution spectrale pour suivre l’etat des couverts vegetaux In: Guyenne, T D, Hunt, J J (Eds ), Spectral Signatures of Objects in Remote Sensing Vol 287 of ESA Special Publication. 1988, pp 279–286.

[46] Huete A. A soil-adjusted vegetation index (SAVI). Remote Sens Environ. 1988;25:295–309.

[47] Pen˜uelas J, Baret F, Filella I. Semiempirical indexes to assess carotenoids chlorophyll-a ratio from leaf spectral reﬂectance. Photosynthetica. 1995;31(2):221–230.

[48] Jordan CF. Derivation of leaf-area index from quality of light on forest ﬂoor. Ecology. 1969;50(4):663–&.

[49] Gitelson AA, Merzlyak MN. Remote estimation of chlorophyll content in higher plant leaves. Int J Remote Sens. 1997;18(12):2691–2697.

[50] McMurtrey JE, Chappelle EW, Kim MS, Meisinger JJ, Corp LA. Distinguishing nitrogen-fertilization levels in-ﬁeld corn (Zea mays L) with actively induced ﬂuorescence and passive reﬂectance measurements. Remote Sens Environ. 1994;47 (1):36–44.

[51] Chappelle EW, Kim MS, McMurtrey JE. Ratio analysis of reﬂectance spectra (rars) An algorithm for the remote estimation of the concentrations of chlorophyll-a, chlorophyll-b, and carotenoids in soybean leaves. Remote Sens Environ. 1992;39(3):239–247.

[52] Zarco-Tejada PJ, Miller JR. Land cover mapping at BOREAS using red edge spectral parameters from CASI imagery. J Geophys Res Atmos. 1999;104 (D22):27921–27933.

[53] Lichtenthaler HK, Lang M, Sowinska M, Heisel F, Miehe JA. Detection of vegetation stress via a new high resolution ﬂuorescence imaging system. Plant Physiol. 1996;148(5):599–612.

[54] Hern´andez-Clemente R, Navarro-Cerrillo RM, Zarco-Tejada PJ. Carotenoid content estimation in a heterogeneous conifer forest using narrowband indices and PROSPECT + DART simulations. Remote Sens Environ. 2012;127(0):298–315.

[55] Pen˜uelas J, Filella I, Lloret P, Mun˜oz F, Vilajeliu M. Reﬂectance assessment of mite eﬀects on apple trees. Int J Remote Sens. 1995;16(14):2727–2733.

[56] Elvidge CD, Chen ZK. Comparison of broad-band and narrow-band red and near-infrared vegetation indexes. Remote Sens Environ. 1995;54(1):38–48.

[57] Filella I, Pen˜uelas J. The red edge position and shape as indicators of plant chlorophyll content, biomass and hydric status. Int J Remote Sens.1994;15(7):1459–1470.

[58] Levin N, Kidron GJ, Ben-dor E. Surface properties of stabilizing coastal dunes: Combining spectral and ﬁeld analyses. Sedimentology. 2007;54(4):771–788.

[59] Lobell DB, Asner GP, Law BE, Treuhaft RN. Subpixel canopy cover estimation of coniferous forests in Oregon using SWIR imaging spectrometry. J Geophys Res. 2001;106(D6):5151–5160.

[60] Hunt ER, Doraiswamy PC, McMurtrey JE, Daughtry CST, Perry EM, Akhmedov B. A visible band index for remote sensing leaf chlorophyll content at the canopy scale. Int J Appl Earth Obs. 2013;21:103–112.

[61] Broge N, Leblanc E. Comparing prediction power and stability of broadband and hyperspectral vegetation indices for estimation of green leaf area index and canopy chlorophyll density. Remote Sens Environ. 2001;76(2):156–172.

[62] Vogelmann JE, Rock BN, Moss DM. Red edge spectral measurements from sugar maple leaves. Int J Remote Sens. 1993;14(8):1563–1575.
